# A correlative and quantitative imaging approach enabling characterization of primary cell-cell communication: Case of human CD4^+^ T cell-macrophage immunological synapses

**DOI:** 10.1038/s41598-018-26172-3

**Published:** 2018-05-22

**Authors:** Richard Kasprowicz, Emma Rand, Peter J. O’Toole, Nathalie Signoret

**Affiliations:** 1 Centre for Immunology and Infection, Department of Biology and Hull York Medical School, University of York, YO10 5DD York, United Kingdom; 2 Department of Biology, University of York, YO10 5DD York, United Kingdom; 3 Bioscience Technology Facility, Department of Biology, University of York, YO10 5DD York, United Kingdom

## Abstract

Cell-to-cell communication engages signaling and spatiotemporal reorganization events driven by highly context-dependent and dynamic intercellular interactions, which are difficult to capture within heterogeneous primary cell cultures. Here, we present a straightforward correlative imaging approach utilizing commonly available instrumentation to sample large numbers of cell-cell interaction events, allowing qualitative and quantitative characterization of rare functioning cell-conjugates based on calcium signals. We applied this approach to examine a previously uncharacterized immunological synapse, investigating autologous human blood CD4^+^ T cells and monocyte-derived macrophages (MDMs) forming functional conjugates *in vitro*. Populations of signaling conjugates were visualized, tracked and analyzed by combining live imaging, calcium recording and multivariate statistical analysis. Correlative immunofluorescence was added to quantify endogenous molecular recruitments at the cell-cell junction. By analyzing a large number of rare conjugates, we were able to define calcium signatures associated with different states of CD4^+^ T cell-MDM interactions. Quantitative image analysis of immunostained conjugates detected the propensity of endogenous T cell surface markers and intracellular organelles to polarize towards cell-cell junctions with high and sustained calcium signaling profiles, hence defining immunological synapses. Overall, we developed a broadly applicable approach enabling detailed single cell- and population-based investigations of rare cell-cell communication events with primary cells.

## Introduction

Contact-based cell communication is an essential mode of crosstalk in the functioning of mammalian organs, tissues, nervous and immune systems. Understanding the molecular basis of functional cell connections is therefore important to tackle diseases resulting from their breakdown. Insights require techniques to visualize and characterize these connections in action, many of which have been developed to investigate immune communication. Cells of the immune system can form specialized cell-cell junctions defined as immune synapses and kinapses (IS and IK), two differing modes of signaling interactions that are interchangeable but remain functionally distinct^1–6^. IS is a stable signaling junction marked by the formation of micrometer scale three-dimensional domains of signaling components, cell surface proteins and lipids that accumulate to the cell interface^3,7–9^. Our knowledge of the organization and dynamics of these junctions comes from *in vitro* or *in vivo* studies using genetically modified cells over-expressing labeled cellular markers to track conjugates formed between a T cell and an antigen-presenting cell (APC)^6,10–13^. These relatively targeted experimental approaches are tailored for investigations of known or predicted events associated with already characterized T cell-APC conjugates^6,10–13^. However, conjugates are highly regulated and dictated by the subsets, activation status and combination of cell involved, as well as the type of antigen presented^2,14,15^.

The study of previously uncharacterized conjugates involving different cell-types within complex cell populations or experimental systems requires a different approach. All cell conjugate forming events must be identified, together with a readout of signal productive cell-cell engagement that is independent of junction types. These are essential to determine the frequency and occurrence of these junctions in an unbiased manner, even before defining interactions to be assessed for dynamics and organization. Detecting a rise in cytoplasmic calcium is a suitable broad-spectrum readout of cell-cell communication and of crosstalk between immune cells^16^. Real-time imaging of intracellular calcium flux is also a validated method to identify single signaling T cells in primary cell populations where these can represent relatively rare events^17^. Therefore, monitoring T cell calcium from conjugates formed over time would allow for unbiased identification of any productive antigen-dependent T cell-APC interactions, regardless of the subsets and combination of cells involved, the frequency, stability or duration of the interaction. Live image recordings can be used to characterize calcium profiles and dynamics of interacting T cells^17,18^, whilst subsequent staining of the imaged samples can inform on molecular events occurring in signaling conjugates. To gain a more in depth understanding of what defines IS formation, data from large numbers of conjugates need to be acquired, quantitatively and statistically analyzed. Combining these methods in a correlative approach using calcium-sensitive reporter dyes and detection of endogenous cell markers would allow single cell- and population-based investigations of cell-cell junctions, even with primary cells.

To test the validity of such an approach, we investigated an uncharacterized form of T cell-APC junction between human CD4^+^ T cells and macrophages in primary cell cultures. Antigen-dependent interaction of macrophages with CD4^+^ T cells forms an important aspect of cell-mediated immunity that can result in macrophage activation. *In vitro*, IS or IK formation between murine macrophage lines and transformed T cells has been shown to be highly dynamic^19^. However, direct visualization of this type of junction and whether typical markers accumulate to the cell-cell interface has not yet been demonstrated in primary cells. Peripheral blood mononuclear cells (PBMCs) are convenient to investigate diversity in immune cell interactions because non-clonal subpopulations of T cells and autologous APCs can be isolated, activated and/or differentiated *in vitro*. Various forms of T cell/APC conjugates have been reported using PBMCs and described by confocal microscopy or imaging flow cytometry^20–23^. By adding live calcium imaging and recording to correlated confocal imaging of cell conjugates, we deliver an improved method, focusing on the formation of functional cell-cell interactions.

Overall, we describe an accessible approach that can deliver high throughput qualitative and quantitative assessments of rare dynamic events to obtain insights into the spectrum of antigen dependent cell-cell interactions between populations of immune cells and identify those that represent functional bona fide synapses. Since calcium signaling is a relatively ubiquitous readout of cell excitability, this approach can be extended to other cellular systems and more general studies of cell-cell communication.

## Results

To account for the diversity of antigen-dependent interactions, we chose to assess the interaction of human MDMs with autologous CD4^+^ T cells in presence of bacterial superantigen (sAg). Staphylococcal sAgs, enterotoxins A, B and/or E (SEA, SEB, SEE), stabilize immune cell-conjugates by crosslinking Major Histocompatibility Complex class II (MHCII) molecules on APCs to T cell receptors (TCR) for up to 20% of the peripheral blood T cell repertoire^24^. This scenario creates diversity in the experimental situation, as only a subset of T cells will be engaged in stabilized antigen-dependant interactions.

### Assessing the frequency of signal productive conjugates within a population

**Figure 1:**
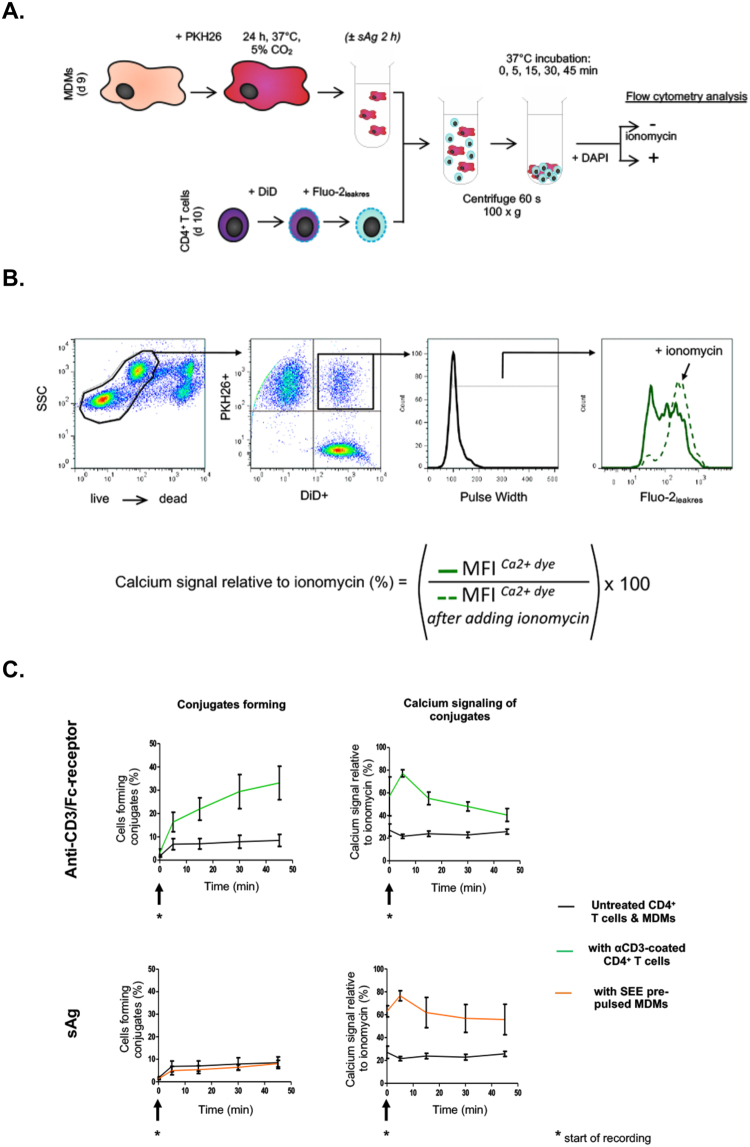
Flow cytometry strategy to assess the frequency and signaling of MDM-CD4+ T cell conjugates. (**A**) Schematic diagram describing the experimental protocol developed; MDMs labeled with the lipophilic membrane dye PKH26 were exposed or not to sAg (1 µg/ml SEE), before incubation with Fluo-2_leakres_ loaded CD4^+^ T cells pre-labeled with a distinct lipophilic membrane dye (DiD); DAPI stained samples were exposed or not to ionomycin and analyzed by flow cytometry (>10,000 events acquired per sample). (**B**) Gating strategy and output metrics. Pulse Width gated Fluo-2_leakres_ Mean Fluorescence Intensity signal (MFI; green bold line) was expressed as a percentage of the signal within the same gate after adding ionomycin to the sample (green dotted line). (**C**) Graphs showing cell conjugate formation over time and the intensity of calcium signals within these conjugates, for T cells pre-coated with an anti-CD3 antibody (OKT3, 5 µg/ml; Anti-CD3/Fc receptor panel), presented to SEE pre-pulsed MDMs (1 µg/ml; sAg panel) or to untreated MDMs (black lines). Each graph represents the mean (±SD) from experiments performed on cells from N = 3 donors.

A time-staggered flow cytometry protocol was developed to investigate the extent to which addition of sAg affects the proportion of T cell–APC conjugates forming over time, and T cell signaling within these conjugates. The protocol involved labeling live MDMs and CD4^+^ T cells with lipophilic membrane dyes, while loading T cells with a calcium sensitive reporter dye (described in Fig. 1A**)**. This protocol makes effective use of the high sampling rate of a flow cytometry platform to analyze a large population of cells, from which the frequency of CD4^+^ T cell-MDM conjugate formation can be determined by gating on events positive for both lipophilic dyes (PKH26^+^DiD^+^ cells, Fig. 1B). Loading of T cells with Fluo-2_leakres_ allowed for measurements of antigen-dependent signaling within the CD4^+^ T cell-MDM conjugates formed. At each time point, samples were acquired to detect Fluo-2_leakres_ fluorescent signal before and after addition of ionomycin. Levels of intracellular calcium signals within the population of conjugates formed could be expressed as a fraction of the reference maximum signal released by ionomycin (Fig. 1B).

Suitability of this protocol was tested with an established positive control:^25,26^ anti-CD3-coated CD4^+^ T cells were mixed with MDMs to trigger anti-CD3/Fc-receptor mediated CD4^+^ T cell/MDMs conjugates with T cell signaling (Fig. 1C). An increase in double positive PKH26^+^DiD^+^ calcium signaling events over the 45 min timeframe of the assay was apparent, confirming the ability of the method to specifically detect the formation of signaling cell conjugates. We then applied this protocol to examine the effects of sAg on antigen-dependent CD4^+^ T cell-MDM conjugate formation (Fig. 1C). In the absence of sAg, CD4^+^ T cell-MDM conjugates formed at a low frequency (mean ± sd: 8 ± 3%), which plateaued after a 5 min co-incubation period. T cell incubation with MDMs pre-pulsed with the sAg SEE showed a similar low frequency (8 ± 2%). However, only SEE treatment was accompanied with a rise in calcium signal from conjugates, which peaked in intensity after 5 minutes, as seen with anti-CD3/Fc-receptor mediated conjugates (Fig. 1C). These experiments show that *in vitro* formation of conjugate between autologous blood-derived human CD4^+^ T cells and MDMs is infrequent and not augmented by sAg, but that sAg-dependent CD4^+^ T cell-MDM interactions trigger calcium signaling within the conjugate population.

### Developing an image-based assay capable of identifying productive CD4^+^ T-MDM conjugates

**Figure 2:**
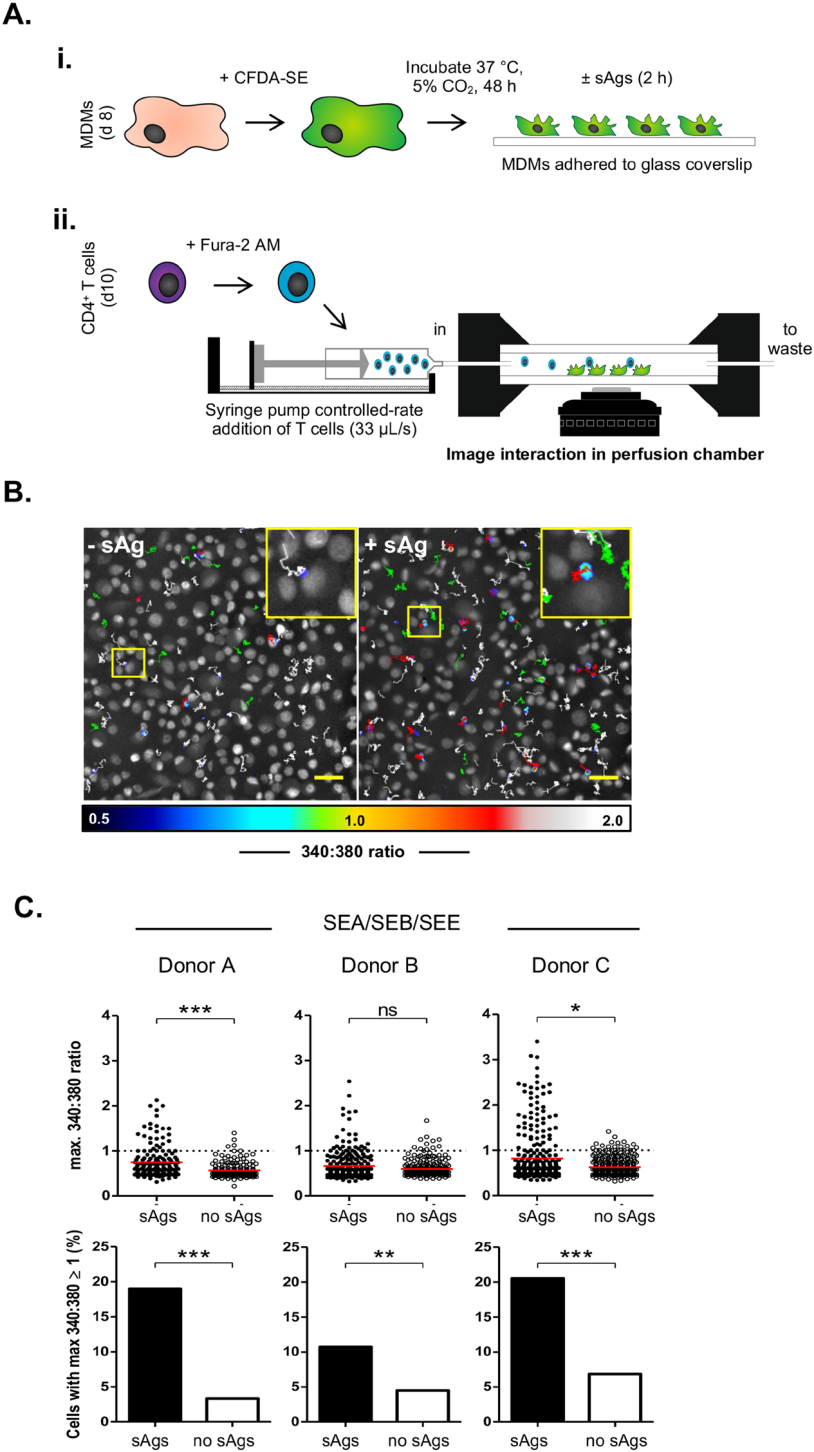
Imaging CD4^+^ T cell signaling in the context of MDM-CD4^+^ T cell conjugates. (**A**) A cartoon diagram of the live imaging strategy: (i) CFDA-SE labeled MDMs seeded on glass coverslips were pulsed or not with sAg, (ii) before recording calcium signals from Fura-2-AM loaded CD4^+^ T cells perfused over MDMs, with an inverted fluorescence microscope. (**B**) Image of the final timeframe of acquisition (t = 20 min) recording changes in intracellular calcium signals (340:380 ratio) from T cells added to MDMs (white cells) treated or not with sAg (1 µg/ml SEE). Individual T cells were tracked using MTrackJ and tracks were colored according to the maximum 340:380 ratio experienced over the course of the assay (green ≤0.5, white ≥0.5 and ≤1.0; red ≥1.0). Insets are zoomed views of yellow boxed-regions; scale bars = 50 µm. (**C**) Similar experiments carried out using cells from three donors and MDMs pulsed with a mixture of sAgs (SEA/SEB/SEE); for each donor, results are expressed for the maximum 340:380 ratio for every cell in each field of views and data were compared using a Mann-Whitney U test (beeswarm plots: red bar, median; dotted line, 340:380 threshold level) or by plotting the frequency of events acquired, which fall above the 340:380 threshold level (bar graphs: data compared using Fisher’s exact test).

Flow cytometry protocol provided a Boolean measure for occurrence of antigen-dependent interactions and a measure of their frequency within mixed cell populations, through rapid sampling of over 10,000 events per samples. However, the next steps involved determining the nature of these cell-cell interactions, which requires measurements of temporal events of individual cells and assessment of the spatial arrangement of proteins at the cell-cell interface. Since such measurements cannot be determined via traditional flow cytometry, we chose to adapt our approach for live cell wide-field fluorescence microscopy. We used the prior flow cytometry results to inform development of a protocol for imaging interactions between CFSE-labeled MDMs and CD4^+^ T cells pre-loaded with the ratiometric intracellular calcium indicator Fura-2/AM (Fig. 2A). The assay was conducted at high cell densities (107 ± 48 CD4^+^ T cells and 198 ± 38 MDMs [mean ± SD] per 470 × 470 µm region) due to the expected 8% frequency of conjugate-forming events. Based on the fast onset of sAg-dependent calcium signaling detected by flow cytometry, we opted for a controlled rate injection of T cells over MDMs in a perfusion chamber with immediate imaging initialization and use of an imaging interval of 10 s. A color scale was applied to Fura-2/AM 340:380 intensity ratio time-lapse images, to indicate the cytoplasmic calcium levels of CD4^+^ T cells during interactions with MDMs pre-pulsed or not with sAg (Fig. 2B**)**. The extended duration of red or white flashing T cells in time-lapse images provides an immediate visual indicator that more T cells exhibit high levels of intracellular calcium in the presence of sAg (Mov 1–3). To quantify the extent of intracellular calcium and T cell migration, T cells were tracked over the assay time course with measures of 340:380 ratios extracted for individual cell. The maximum 340:380 ratio (340:380_max_) was used as an immediate readout of the amplitude of calcium signaling for each CD4^+^ T cell within the imaged region (Fig. 2C).

Initially, imaging experiments were carried out using only SEE (Fig. S1), but to improve donor-to-donor consistency in T cell responses, subsequent assays were conducted using a cocktail of three sAgs (SEA, SEA and SEE) to broaden the spectrum of TCR Vβ recognized by the sAg, as previously reported^27^. Analysis of the entire T cell population imaged includes contributions of both T cells engaged in conjugates and those existing in isolation (Fig. 2C, beeswarm plots). Any non-significant differences between T cell calcium responses ±sAgs reflect the low frequency of conjugate formation. Sensitivity to events with low frequency within the population requires exclusion of the basal CD4^+^ T cell calcium signaling events (340:380_max_ ratio ≤ 1) that occur for the majority of non-conjugated CD4^+^ T cells. This was done by applying a threshold (340:380_max_ ≥ 1) to the analysis (Fig. 2C, bar graphs). Fisher’s exact test of the number of events above threshold revealed that the number of CD4^+^ T cells responding with calcium levels ≥ 1 was significantly greater for all donors when cells were introduced into MDM cultures pulsed with sAgs (Fig. 2C, bar graphs). Thus, we have established an imaging assay and analysis routine with sufficient sensitivity to immediately detect sAgs-dependent calcium signaling events that occur at a low frequency between a heterogeneous, polyclonal co-culture of MDMs and autologous CD4^+^ T cells derived from human blood.

### Single-cell analysis of CD4^+^ T cell calcium signatures

**Figure 3:**
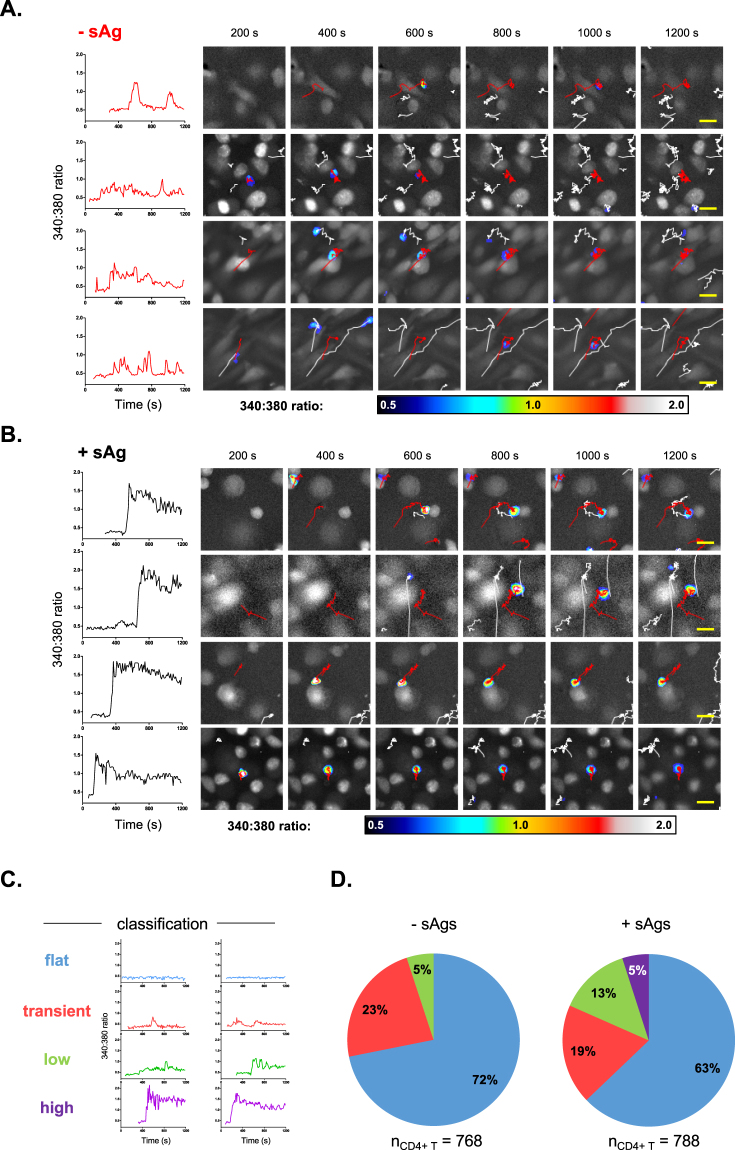
Single cell analysis of time-lapse images reveals that CD4^+^ T cells exhibit distinct intracellular calcium signatures when interacting with sAgs-pulsed MDMs. (**A** and **B**) Intracellular calcium profiles of CD4^+^ T cells recorded with a 340:380 ratio exceeding the threshold value (≥1); plots show the intracellular calcium fluxes of CD4^+^ T cells over time, when perfused over untreated (**A**) or sAgs-pulsed (**B**) MDMs; the MDM (large white cell) interacting CD4^+^ T cell considered in each plot is visible in the adjacent image frames, highlighted with a red track and labeled according to the 340:380 color-scale. (**C**) Calcium signatures of CD4^+^ T cells on MDMs were grouped into four categories according to their 340:380 ratio profiles, as illustrated with representative plots. (**D**) Charts indicating the percentage of CD4^+^ T cell calcium signatures classified into each group in the presence and absence of sAgs. Scale bars = 20 µm.

Up to now, CD4^+^ T cell-MDM interactions have been considered on the population level. All CD4^+^ T cell intracellular calcium responses have been measured according to the maximum level of calcium experienced by each cell throughout the assay. Yet the image-based assay provides a temporal axis over which intracellular calcium levels of individual CD4^+^ T cell can be plotted. Each plot, which we term a calcium signature, can then be considered on a single-cell basis. Examples of calcium signatures that occurred within untreated and sAgs-pulsed MDM samples are shown in Fig. 3A and B, respectively. All the signatures depicted come from T cells designated to belong to the acute calcium-signaling group (340:380_max_ ≥ 1), as defined in the population-type analysis. Despite all the signatures shown being grouped together, clear differences in temporal aspects of calcium signaling are apparent when viewed as single-cell signatures. In the absence of sAgs, calcium profiles of CD4^+^ T cells show transient rises that return to baseline levels within the 20 min imaging timeframe (Fig. 3A). In contrast, when MDMs are pulsed with sAgs, the calcium profiles of CD4^+^ T cells often have sharp, high-amplitude onsets that maintain a high level for the remainder of the imaging timeframe (Fig. 3B).

In order to better define profiles associated with sAgs-pulsed MDMs, calcium signatures were visually classified into four groups ‘*flat’*, ‘*transient’*, ‘*low’* and *‘high’* (Fig. 3C), as defined in material and methods. In presence and absence of sAgs, the majority of CD4^+^ T cell calcium signatures were classified as ‘*flat’* (63%, +sAgs; 72%, −sAgs), indicating that these cells do not engage with MDMs in a manner that instigates calcium signaling (Fig. 3D). In the absence of sAgs, ‘*transient’* signatures predominated in responding CD4^+^ T cells. Whilst a similar percentage of ‘*transient’* profiles existed in the presence or absence of sAgs, the frequency of ‘*low’* but stable calcium profiles increased with sAgs-pulsed MDMs. Most importantly, the addition of sAgs led to the appearance of a unique *‘high’* group of CD4^+^ T cell calcium profiles (Fig. 3D). These data indicate that CD4^+^ T cell-MDM interactions in presence of sAgs not only causes high-amplitude calcium responses but also expands the number of CD4^+^ T cells that engage in lower-amplitude sustained calcium signaling.

### Multiparametric space analyses identify CD4^+^ T cell ‘responders’ in conjugates

**Figure 4:**
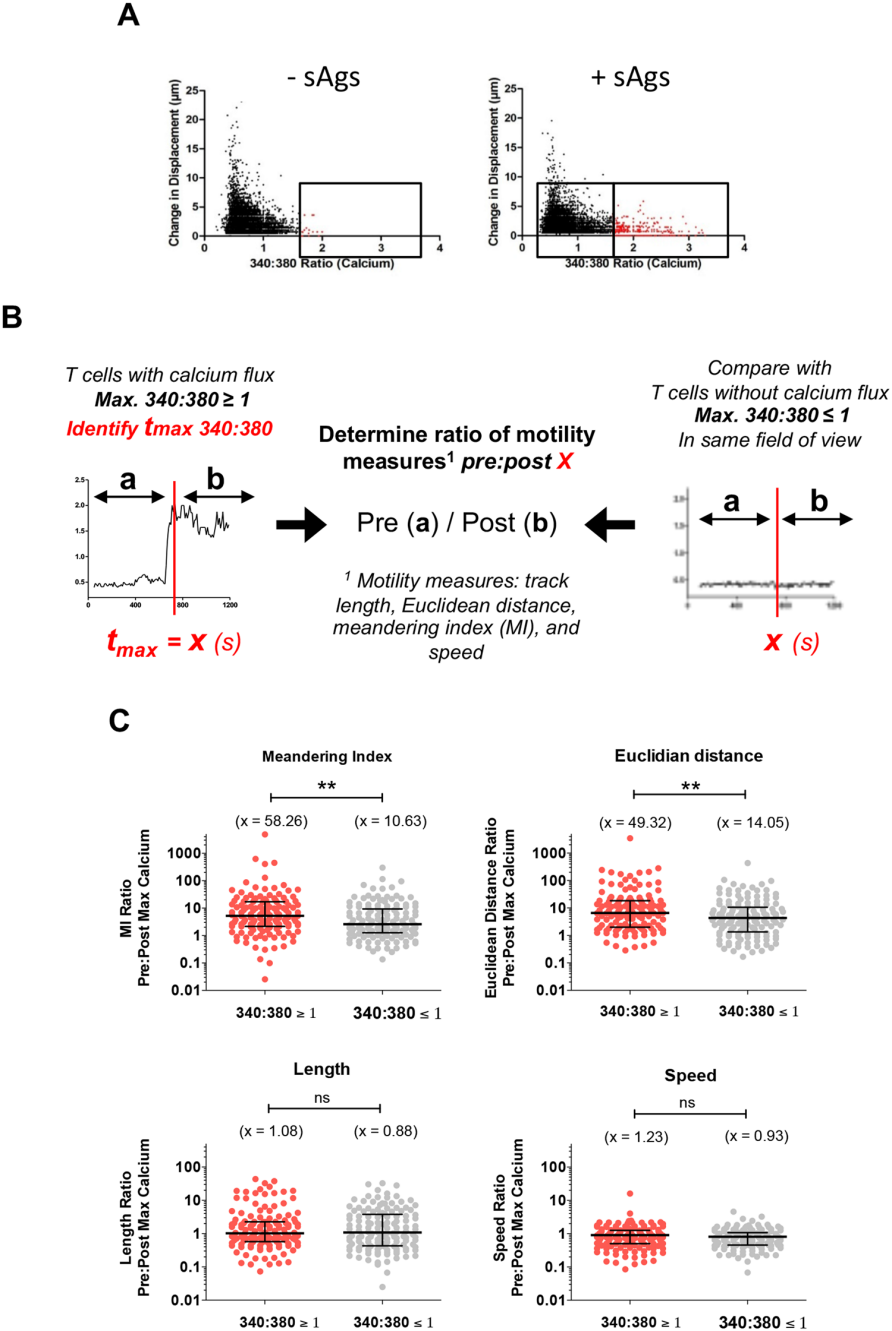
Analysis of the change in motility parameters either side of the maximum calcium signal tracked. (**A**) CD4^+^ T cells were perfused over sAgs-spiked or untreated MDMs. Scatter plots show individual “objects” (T cells) identified in every image frame from live imaging experiments with cells from one donor. The change in displacement measures how far that object has moved since the previous time frame. Objects with 340:380 ratios greater than two standard deviations above the mean (i.e. ratios >1.636) of maximum calcium intensities are highlighted in red. **(B)** Diagram of the pre:post max 340:380 ratio analysis adopted to determine differences in motility before and after calcium flux within a background of a flow rate that decreases over time and can differ between regions imaged in different lateral positions. **(C)** Comparison of meandering index (MI) ratio, Euclidean distance ratio, length ratio and speed ratio before and after the max 340:380 signal. Each point represents an individual cell measured from donor (n = 3) samples treated with sAgs [Mean, x; Bars: median ± interquartile range. Statistical analysis: Mann Whitney U test].

Time-lapse images ±sAgs show that a fraction of T cells undergo calcium signaling with concurrent restriction of migration, whilst in close proximity to an MDM (Mov. 4). Plots of frame-to-frame displacement against 340:380 calcium ratio for all time points within every T cell track show that restriction of migration is not uniquely associated with increased calcium signaling (Fig. 4A); albeit acute calcium signals (340:380_max_ ≥ 1) only occurred in T cells exhibiting changes in displacement <5 µm/frame. We sought to identify whether the onset of calcium signaling, defined as the time of 340:380_max_ (t_340:380max_), is followed by any significant changes in the following T cell motility measures: track length, Euclidean distance, meandering index (Euclidean distance/track length), and speed. The t_340:380max_ for each T cell recorded as undergoing acute calcium signaling (340:380_max_ ≥ 1) was matched to the cell with the closest t_340:380max_ within the T cell population undergoing basal calcium signaling (340:380_max_ ≤ 1). Next, the ratio of the total motility measure [pre-t_340:380max_]:[post-t_340:380max_] was calculated (Fig. 4B). Performing t_340:380max_ matching and determining the ratio of each motility measure on a per-cell basis accounts for the gradual slowing in fluid flow after initial perfusion of T cells and for positional variation in the speed of fluid flow within the perfusion chamber. Analysis of ratio motility measures between T cells from acute and basal calcium signaling groups within sAg-pulsed samples showed statistically greater Pre/Post t_340:380max_ ratios of Euclidean distance and meandering index for T cells undergoing acute signaling (Fig. 4C). This equates to reduced Euclidean distance but not track length or speed in response to calcium signals. Such differences were not detected when comparing conjugates formed without sAgs (Fig. S2). Overall, our motility analysis indicates that CD4^+^ T cells engaged with MDMs in a sAg-dependent manner do not simply stop migrating but become corralled to a position close to the initial site of calcium signal onset.

**Figure 5:**
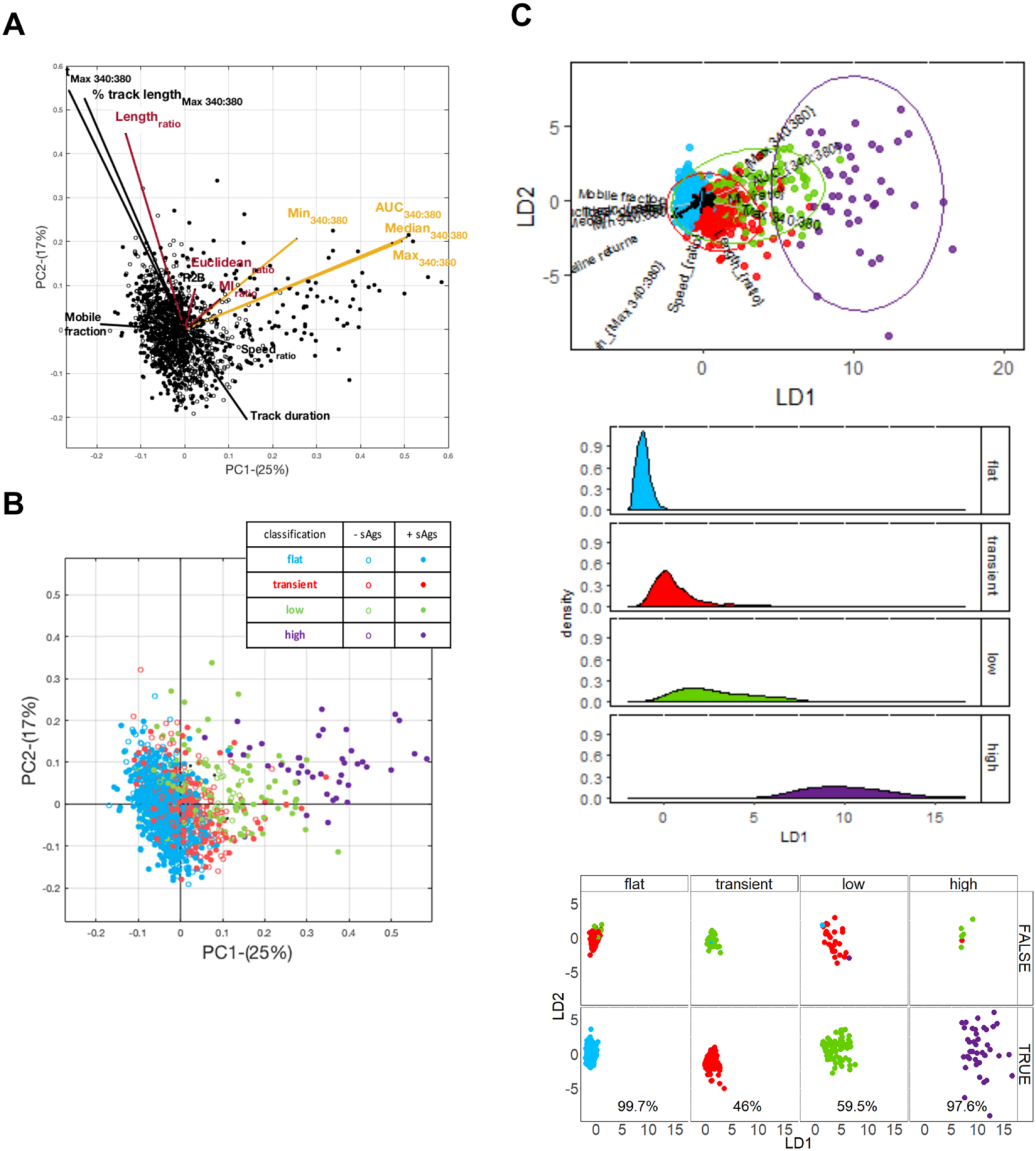
Multivariate data analysis: Observations (n = 1391) for 13 measures of T cell responses ±sAgs projected on to the first two principal components (**A** and **B**) and linear discriminants (**C**). (**A**) PCA bi-plot projecting observations from sAgs-treated (closed symbols) and untreated (open symbols) samples; lines indicate the contribution of each measure to the two axes. (**B**) PCA bi-plot projecting the same observations according to the visual calcium classification. (**C**) LDA assessing calcium signatures separation (*‘Flat’* blue, *‘transient’* red, *‘low’* green, *‘high’* purple): Bi-plot of the first two LD scores identifying the calcium profile groups with 95% confidence ellipses for individual groups (top panel); density plots of the first linear discriminate (middle panel) and class prediction plots (bottom panel) demonstrate the reliability of classification to the ‘flat’ and ‘high’ categories of calcium signatures; percentage of predictions that agree with visual classification is given.

To consider the relative importance of motility and calcium signature measures extracted for each T cell in discriminating between individual CD4^+^ T cell responses, all measures for all donors were considered together in multiparametric space analyses (Fig. 5). Principal Component analysis (PCA) looked for the direction showing the largest total variance between parameters (Figs 5A,B and Mov. 5). Bi-plots of the first two principal component (PC) scores (Figs 5A,B**)**, or three PCs in a 3D version (Mov. 5) showed points spreading away from the main population clustered around the origin of the axes. The vector lines indicated that the highest magnitude contributions to the spread come from calcium dependent measures (AUC_340:380_, Max_340:380_, Median_340:380_, Min_340:380_), with only minor impact from motility measures (Fig. 5A and Mov. 5). To determine whether unsupervised classification might be used to define CD4^+^ T cell responses, the groups defined by visual classification were highlighted in color on a two PC bi-plot (Fig. 5B). Samples visually classified as ‘*flat’* and ‘*transient’* profiles were concentrated around the axes’ origin, while ‘*low’* profiles +sAgs spread from the PC1 origin, and *‘high’* profiles only seen with sAgs clustered as a separate population of ‘responders’ away from all origins (Fig. 5B and Mov. 5). To ascertain the relevance of our visual calcium classification in separating out ‘responders’ we used Fisher’s Linear Discriminant Analysis (LDA), a supervised statistical method that maximizes the separation between groups^28^. LDA identified the different calcium signature groups (Fig. 5C, bi-plot) showed complete separation on the first linear discriminant for the ‘*flat’* and *‘high’* groups, but overlap for *‘transient’* and *‘low’* signatures, suggesting less accuracy in allocating intermediate profiles (Fig. 5C, LD1 density plots). There was very high correspondence between predicted and observed *‘high’* profiles that defined T cell ‘responders’, with 97.6% of cases correctly classified (Fig. 5C, class prediction plot). These analyses confirm that quantitative parameters derived from calcium cell tracking can discriminate CD4^+^ T cells responding to MDMs pulsed with sAgs.

### Calcium signatures that correspond to the formation of the CD4^+^ T cell –MDM immune synapse

**Figure 6:**
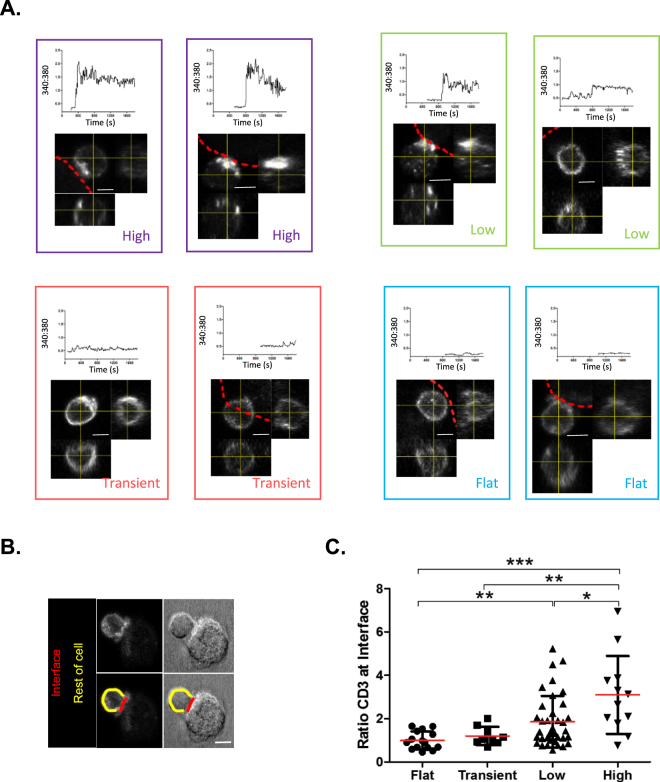
Correlative imaging approach to assess CD4^+^ T cell calcium signatures from immune synapses. Following live recording of intracellular calcium, samples were fixed, permeabilized and stained to identify synapses by accumulation of CD3 at the MDM-CD4^+^ T cell interface. A pre-defined point of reference on the glass-bottom dish help to mark out the position of the original field of view to Z stack confocal images, and allows correlation between the intracellular calcium profile and CD3 capping on individual CD4^+^ T cells. (**A**) Calcium profiles (classified into the indicated group) and their corresponding orthogonal views of CD3 accumulation at the MDM-CD4^+^ T cell interface. The leading edge of the interacting MDM is indicated by the red dotted line; scale bars = 5 µm. (**B**) Diagram of the method used to determine accumulation ratio of the CD3 marker to the cell-cell interface. (**C**) Accumulation of CD3 occurs within each class of calcium signatures, but the accumulation ratio significantly increases with the intensity of the calcium signal. Experiments were performed on cells from N = 4 donors; statistical comparison was performed using ANOVA followed by a Tukey post-hoc test.

To assess which calcium signature(s) relate to CD4^+^ T cell-MDM immunological synapse formation, calcium signaling of cell conjugates was recorded for 30 min prior to fixation and immunolabeling with anti-CD3 for correlated imaging. Confocal Z-stacks were acquired of cells in the original regions used for live calcium recording, and CD3 3D distribution linked to the calcium signatures on a single-cell basis. The accumulation of endogenous CD3 to the interface of sAgs-dependent cell-cell conjugates marked IS formation (Fig. 6**)**. Orthogonal images showed the accumulation of CD3 correlating with cells exhibiting sustained calcium signatures (*‘high’* and ‘*low’*), whilst CD3 appeared to be evenly distributed across the surface of T cells with basal calcium signals (‘*transient’* and ‘*flat’*; Fig. 6A**)**. The ratio of CD3 at the CD4^+^ T cell-MDM interface compared to the rest of the T cell was determined to quantify its accumulation (Fig. 6B). Quantification confirmed that CD3 accumulation to synapse interface only occurs in CD4^+^ T cells that have experienced ‘*high*’ or ‘*low*’ calcium signatures (Fig. 6C). Interestingly, not all ‘*low’* calcium signatures result in measurable CD3 accumulation at the cell-cell interface, suggesting a mix of either kinapses or recently formed synapses^6,16^. Note that the fixation process eliminates free or weakly interacting CD4^+^ T cells and increases the apparent proportion of cells with ‘*high*’ or ‘*low’* calcium (Fig. S3). Combining assessments of intracellular calcium and CD3 distribution together, led us to capture rare incidences of bona fide immune synapses formed.

### Analysis of endogenous proteins localizing to the interface of productive CD4^+^ T cell-MDM synapses

**Figure 7:**
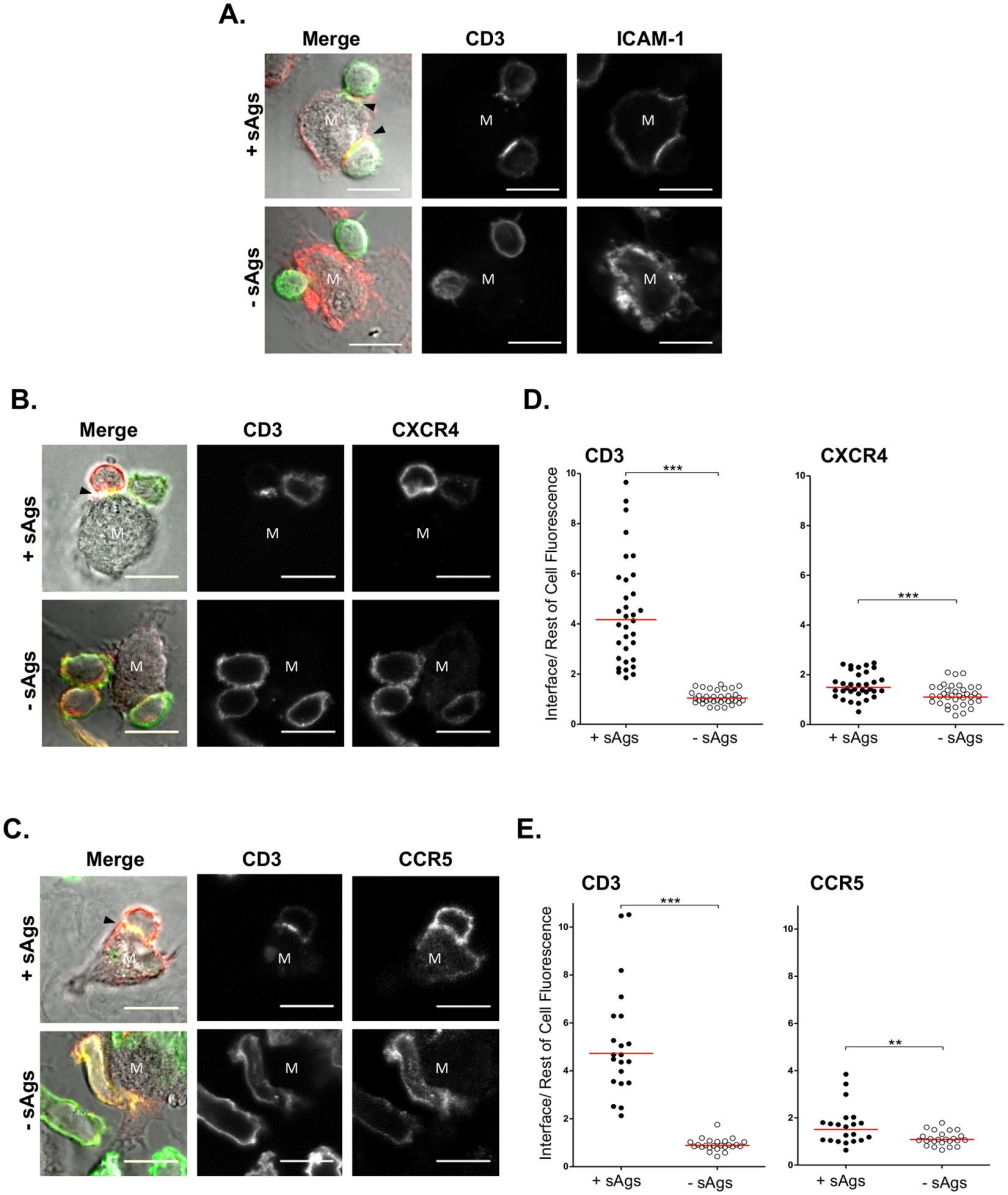
Adhesion proteins, and surface chemokine receptors are recruited to the MDM-CD4^+^ T cell interface within synapses. Immunolabeling of intact MDM-CD4^+^ T cell conjugates formed ±sAgs. T cell surface distribution of CD3 with ICAM-1 from MDMs (**A**), CXCR4 (**B**), or CCR5 (**C**); synapses are identifiable by capping of T cell CD3 (black arrowheads in merge panels) at the interface with the interacting MDM (M). Quantification of the accumulation of CD3 and CXCR4 (**D**) or CD3 and CCR5 (**D**) to the cell-cell interface compared to the rest of the T cell surface; a minimum of 21 conjugates from N = 2 donors were recorded and a ±sAgs comparison was performed using Student’s unpaired t-test; the red bar shows the geometric mean.

**Figure 8:**
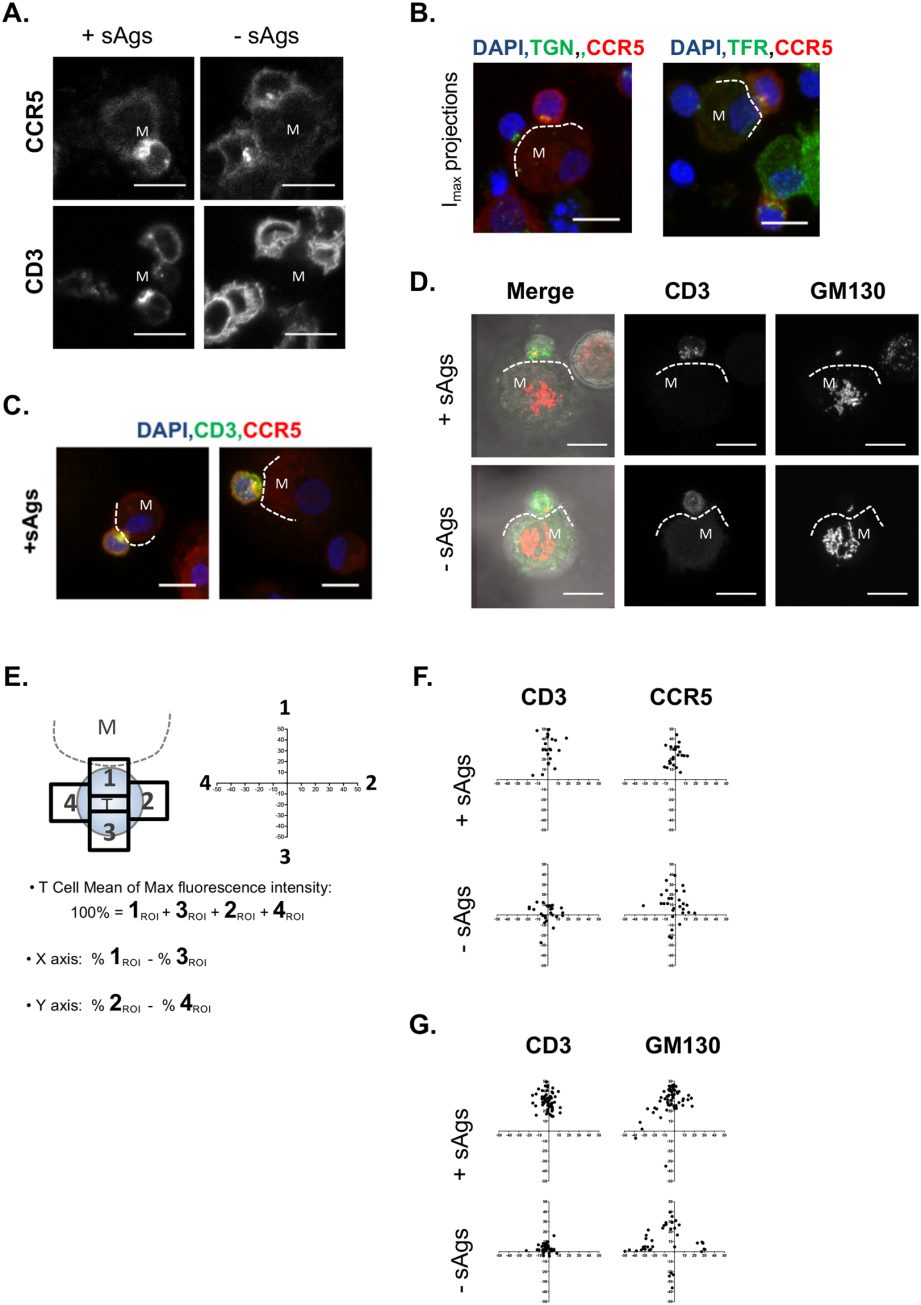
Polarization of cell surface and internal markers from CD4^+^ T cell towards the MDM-CD4^+^ T cell interface. Confocal fluorescence images of MDM-CD4^+^ T cell conjugates formed ±sAgs and immunolabeled after permeabilization; the edge of the interacting MDM (M) is identified with a dotted line; scale bars = 10 µm. (**A**) Comparing total CCR5 and CD3 distribution in the presence and absence of sAgs; representative single sections of conjugates imaged from two to three donors. (**B**) Panels of maximum intensity projections (I_max_) for CCR5 and TGN or TfR co-staining. (**C**) Z-stack projection of CD4^+^ T cells-MDM conjugates with sAgs, co-stained for CD3 and CCR5. (**D**) Confocal fluorescence images show CD3 and T cell GM130 polarized towards the interface of cell conjugates stained permeabilized. (**E**) Diagram of the method used to quantify the polarization of total (surface and internal) staining from CD4^+^ T cells in conjugates. (**F**) Plots showing the distribution of total CCR5 and CD3 quantified from at least 23 conjugates ±sAgs (N = 2 donors). (**G**) Distribution of GM130 and total CD3 quantified from 40 and 60 conjugates ±sAgs (N = 2 and 3 donors, respectively).

This was investigated by correlative analysis of calcium recorded in CD4^+^ T cell-MDM synapses co-stained for CD3 and a marker of interest (Figs 7 and 8). On fixed cells labeled intact, the integrin ICAM-1 expressed at the surface of MDMs was observed to accumulate opposite to capped CD3 with sAgs (Fig. 7A), as reported for other synapses^9^. Note that the classical concentric rearrangement of CD3 and ICAM-1 on opposite sides of a synapse could be seen when, in rare occasions, the orientation of a CD4^+^ T cell-MDM conjugate allowed for a planar interface (Fig. S3). Surface protein accumulation alongside CD3 on T cells was then examined. Previous reports have indicated that GFP-fused chemokine receptors CXCR4 and CCR5 ectopically expressed in CD4^+^ T cells accumulated at IS interfaces formed with DCs or B cells^27,29^. Here, we investigated the fate of endogenous CXCR4 or CCR5, the latter being expressed on some but not all CD4^+^ T cells (Fig. S4)^30^. We found that conjugates stained fixed/intact to only detect cell surface CXCR4 and CCR5 molecules showed very marginal changes in their overall distribution on MDM-interacting CD4^+^ T cells, compared to CD3 (Fig. 7B,C). Nevertheless, analysis of the ratio of staining accumulation to the interface (via the method illustrated in Fig. 6B) revealed that for CD4^+^ T cells interacting with MDMs with sAgs, CXCR4 and CCR5 slightly but significantly increased at the interface concomitantly to CD3 (Fig. 7D,E). Although MDMs also express cell surface CCR5^31^, the staining pattern of MDM CCR5 did not change with the formation of conjugates.

Following antigen-dependent IS formation, many molecular rearrangements occur under the plasma membrane of the interacting T cells, such as the polarization of intracellular organelles^32^. Labeling conjugates after permeabilization exposed an intracellular pool of CCR5 close to cell-cell interfaces, even in the absence of sAgs (Fig. 8A). This pool overlapped with the trans-golgi network (TGN) or the Transferrin receptor (TfR), confirming that some CCR5 is intracellular in CD4^+^ T cells, and follows a well-established early recycling route (Figs 8B and S5)^33,34^. In presence of sAgs, CCR5 and CD3 positive vesicles appeared under the cell-cell interface (Fig. 8C), suggesting internalization events known to take place after IS formation^35^. The position of the Golgi apparatus was examined for conjugates co-stained for CD3 and GM130 after fixation and permeabilization. As for CCR5 (Fig. 8A) examples of Golgi apparatus polarized towards conjugate interfaces were visible ±sAgs (Fig. 8D). We modified the quantitative method of analysis described in Fig. 6B to assess the total distribution of cellular markers including surface and internal localization (Fig. 8E). These analyses revealed that sAgs promoted polarization of the T cell pool of CCR5 and GM130 labelled Golgi towards the interface of CD3 capping synapses (Fig. 8F,G). This set of experiments demonstrate that our approach allows for quantitative detection of subtle endogenous molecular rearrangements consequent to IS formation.

Overall, these experiments show that our methodology has the required sensitivity to investigate the dynamic of cell-cell communication between primary cells, to ascertain the calcium signatures associated with rare events of signal productive interactions in mixed cell populations, and characterize key endogenous spatial molecular rearrangements occurring at the cell-cell interface.

## Discussion

Being able to capture events of cell-to-cell communication in real time to investigate the dynamic molecular events engaged, is leading to advances in our understanding of cellular behaviours. Current techniques are largely based on *in vitro* and *in vivo* live imaging of cells expressing specific fluorescent markers, and relatively inaccessible to investigations using primary cell cultures. Here we developed an approach enabling detailed single cell- and population-based investigations of cell-cell junctions with primary cells, which is based on the detection of a ubiquitous second messenger (intracellular calcium). By combining fluorescence time lapse recording and correlative imaging with multivariate statistical methods of analysis, we deliver unique high throughput and unbiased assessments of rare and dynamic cell-cell interaction events. The approach makes as few assumptions about the nature of the cell engagement as possible. It enables the extent of cell-cell interactions within a heterogeneous, polyclonal culture to be measured using widely available scientific instrumentation to define signal-productive events.

Our method was applied to study the formation of an uncharacterized IS engaging human CD4^+^ T cells and macrophages, using *in vitro* assays of conjugate formation between autologous blood-derived primary cells. Our approach considers population heterogeneity in antigen responses, placing it upstream of the homogenous systems studied by total internal reflection fluorescence and super-resolution microscopy that have advanced our understanding of the complex molecular mechanisms associated with immune cell junctions^11,12,26,36^. Despite lacking the environmental context of *in vivo* methods to measure synapse/kinapse formation^37^, our method shares commonality in measurement of T cell calcium signaling and can be used to supplement such studies. The capacity of flow cytometry to rapidly analyze tens-of-thousands of cells labeled with several lipophilic and calcium sensing dyes^38,39^ enabled an initial reporting of the frequency of stable conjugate formation and the T cell signaling events within this subpopulation of conjugates. This population-based study benchmarked the formation of primary CD4^+^ T cell-MDM conjugates, while a live image-based recording protocol was developed to investigate the signaling activity of CD4^+^ T cells forming conjugates individually. The flow cytometry protocol showed that blood-derived CD4^+^ T cells interacted with autologous MDMs at a similarly low frequency ±sAg, but only sAg-dependent conjugation led to T cell signaling. This is in contrast with studies that used cell lines or single clones of CD4^+^ T cells and described a sAg-dependent increase in conjugate formation with APCs^38,40^. Our live cell imaging experiments recorded all signaling T cell events but supported the flow cytometry in showing that a small but significantly greater proportion of CD4^+^ T cells exhibited high levels of intracellular calcium upon interaction with sAg-pulsed MDMs.

Live calcium recording allowed dynamic analysis of interacting CD4^+^ T cells and showed that onset of intracellular signaling (340:380_max_ ratio ≥ 1) coincided with CD4^+^ T cells arrest on MDMs reflecting stable fully signaling conjugate events. Yet many mobile T cells showed low calcium signals (340:380_max_ ratio ≤ 1), which could correspond to kinapses^19^ and reconcile motile behaviour previously seen in murine macrophage-CD4^+^ T cell interactions *in vitro*^19,41^. To convincingly identify such kinapses, the imaging technique needs to be able to differentiate between stable (<1 µm/min), slowly mobile (1–2 µm/min) and dynamic crawling (1–10 µm/min) states^2^, which requires a higher resolution image. CD4^+^ T cells showed *‘flat’* calcium profiles for most cells analyzed, but where increases in calcium were apparent, variable profiles in terms of intensity and duration were classified as *‘transient’* and more sustained profiles as *‘low’* or *‘high’*. The classification was based on validated systems^17,18,42^, which we have adapted to report upon polyclonal CD4^+^ T cell population responses that have an intrinsically wide range of affinities for antigens. This qualitative analysis revealed that *‘high’* calcium profiles were only associated with conjugates formed in presence of sAgs, while the proportion of *‘low’* profiles increased with addition of sAgs, which suggests a mix of synapses and kinapses according to the model suggested by Kummerow *et al*.^16^. Importantly, onsets of *‘high’* profiles correlated with CD4^+^ T cells close apposition to MDM, and an arrest indicative of stable IS formation. Considering thirteen different motility and calcium related measures of individual T cells in multivariate statistical analyses (PCA and LDA) showed how *‘high’* signatures correlated with the appearance of a separate population of ‘responders’. The unsupervised PCA established calcium signaling as prevalent discriminator of T cell engagement, while LDA indicated our visual classification was less discriminatory for *‘low’* and *‘transient’* events, but nearly fully accurate when allocating *‘high’* profiles. This highlights potential to automate and accurately identify calcium functioning conjugates by using the measurements and unsupervised statistical methods described.

Formation of calcium-productive cell conjugates may not necessarily equate to IS interface reorganization, as evidenced by the lack of large-scale molecular clustering reported in T cells forming kinapses compared to synapses^6^. However, correlating calcium signaling with spatial reorganization of the T cell CD3 marker helped to ascertain the formation of fully functional IS. A simple reference point marking system allowed to create a map linking each T cell labeled with CD3 to the calcium signal recorded for that T cell. This approach showed that the accumulation of CD3 at the interface of conjugates only occurred with sAgs, correlated with a rise in intracellular calcium, and was more pronounced for conjugates with *‘high’* signatures, which confirmed the formation of fully functional IS. Simple correlative CD3 co-staining could then be used as a reference to characterize spatial changes of endogenous cell markers within an IS. In particular, our analysis confirmed the polarization of intracellular compartments in IS, whilst revealing a modest but significant accumulation of endogenous chemokine receptors from T cells towards the interface with MDMs. This validates observations reported in other experimental settings^27,29,43^. Together, our experiments defined essential characteristics of a novel form of IS between primary human CD4^+^ T cells and macrophages.

The approach presented is a simple *in vitro* experimental system utilizing non-genetically modified, heterogeneous primary cell cultures, adapted for dynamic investigations of rare calcium-associated cell-cell interaction events. It brings together qualitative, quantitative, supervised, and unsupervised analysis regimens to aid in understanding diversity in cell-cell communication without *a priori* knowledge, with potential applicability even beyond immunological instances.

## Methods

### Reagents and antibodies

Tissue culture reagents and secondary antibodies [AlexaFluor-488 conjugated goat-anti-mouse (GAM) IgG1, AlexaFluor-647 conjugated GAM IgG2a, and AlexaFluor-488 conjugated donkey-anti-sheep IgG] were purchased from Invitrogen. Primary antibodies used were sheep-anti-human TGN46 (TGOLN2; IgG) from Bio-Rad; mouse-anti-human CCR5 (MC-5, IgG2a^44^) and CXCR4 (12G5, IgG2a^45^), CD3 (UCHT1, IgG1; OKT3, IgG2a) from BioLegend, GM130 (35/GM130, IgG1) from BD Biosciences and TfR (H68.4; IgG1) from Invitrogen. Recombinant *Staphylococcus aureus* enterotoxins and sAgs SEA, SEB and SEE were purchased from Sigma-Aldrich and Toxin Technology Inc., respectively. Other reagents were purchased from Sigma-Aldrich, unless stated.

### Cell isolation and culture

Monocytes were isolated from healthy donor blood leukaphoresis cones (National Blood Service, UK) and differentiated to MDMs in cell culture medium [RPMI 1640 with 2 mM L-glutamine, 10% foetal bovine serum (FBS; PAA), 100 U/ml penicillin, 0.1 mg/ml streptomycin, 25 mM HEPES] with 50 ng/ml M-CSF (PeproTech)^46^. One fifth volume of each cone was used for isolation of autologous human CD4^+^ T cells via RosetteSep human CD4^+^ T cell enrichment cocktail (Stemcell Technologies). CD4^+^ T cells were cultured at a starting density of 2 × 10^6^ cells/ml in medium supplemented with 5 µg/ml phytohaemagglutinin for 3 days, then 7 days culture with 10 U/ml recombinant human interleukin-2 (PeproTech).

### Flow cytometry strategy for assessing conjugate formation

MDMs (day 9) were detached then labelled with 2 µM PKH-26 (Sigma-Aldrich) for 5 min at room temperature (RT). Labeling was quenched with FBS, cells were washed in media and returned to culture at 0.5–1.0 × 10^6^ cells/well in a 6 well plate (Corning) for 24 h. Labeled MDMs were left untreated or pulsed with 1 µg/ml sAg SEE for 2 h at 37 °C in suspension. During this time, CD4^+^ T cells were labelled with 250 nM DiD (Life Technologies) at a density of 1 × 10^6^ cells/ml for 5 min at 37 °C. DiD-labelled T cells were washed and incubated in media at a density of 4 × 10^5^ cell/ml for incubation with 300 nM of Fluo-2 leak-resistant calcium sensitive dye (Fluo-2_leakres_; TEFlabs Inc.), 30 min at 37 °C, 5% CO_2_. Both cells were washed in media before adding T cells to MDMs at a 1:1 cell ratio. Unlabelled control and 6 compensation samples were also prepared, in which cells were co-incubated as described but labelled with either PKH26, DiD, Fluo-2_leakres_, PKH26 and Fluo-2_leakres_, or PKH26 and DiD. MDM-CD4^+^ T cell interactions were synchronized by centrifugation (100 × g for 1 min) and conjugate formation analyzed immediately ( t = 0) or after incubation at 37 °C. Samples were run on a CyAn^TM^ ADP flow cytometer (Beckman Coulter) after addition of 4′,6-diamidino-2-phenylindole (DAPI; 50 µg/ml). For each sample 30 µl of 10 µg/ml ionomycin (Sigma-Aldrich) were added after acquisition, and the sample was re-acquired; analysis was performed using FlowJo software (TreeStar). Dead cells (DAPI^+^) were excluded and the percentage of events within the PKH26^+^DiD^+^ quadrant was used as readout of MDM-CD4^+^ T cell conjugate formation. A pulse-width >100 selection gate was used in order to strictly exclude smaller-sized double positive MDM events that might occur as a result of DiD transfer by phagocytosis. This gate was used along with conjugate (PKH26^+^, DiD^+^) population gates in order to obtain a calcium signal not skewed by false positive single cell-type events exhibiting intermediate DiD fluorescence. The Fluo-2_leakres_ mean fluorescence intensity (MFI) of cells within the DiD^+^ gate from run one was normalised to the maximum calcium signal obtainable, which was determined using an identical gate on a tandem second acquisition of the same sample after ionomycin addition; this calcium signal relative to ionomycin was used as a readout of CD4^+^ T cell signalling in conjugates formed with MDMs. These experiments were performed on cells from N = 3 donors.

### Live cell imaging of MDM-CD4^+^ T cell conjugates

MDMs (day 8) were detached, counted and adjusted to a density of 1 × 10^7^ cells/ml, and labeled with 840 nM CFDA-SE (Life Technologies) in PBS for 15 min at 37 °C. Labeled cells were washed and quenched in medium for 30 min at 37 °C, 5% CO_2_ then seeded onto poly-d-lysine (Sigma-Aldrich) coated glass coverslips (2 × 10^5^ cells/coverslip). After 24 h cells were left untreated or pulsed with 1 µg/ml sAgs for 2 h at 37 °C, 5% CO_2,_ as indicated. Concurrently, autologous CD4^+^ T cells were labelled in medium at 37 °C for 30 min with a ratiometric calcium sensitive dye (5 µM fura-2/AM; Biotium Inc), then resuspended at 1 × 10^6^ cells/ml in phenol-red free media and incubated at RT for 30 min. MDMs on coverslips were washed prior to being affixed into an RC-21BRFS closed perfusion chamber (Warner Instruments) using vacuum grease (Dow Corning). The device was filled with phenol-red free medium and sealed by applying another coverslip to form the upper face of the chamber. Imaging was performed with an incubated inverted epifluorescence microscope (see supplemental material). Experiments were repeated with three technical replicates on cells from N ≥ 3 donors.

### Cell tracking and processing of live cell images

Image analysis was performed using ImageJ^47^, the RatioPlus plugin was used to generate an output ratio image of 340 nm and 380 nm time-sequence 16-bit images. The 32-bit ratio image was set to min/max values of 0.5/2.0, which was then translated to a 16-bit range and false-colored by applying a ‘16 colors’ look-up table. Ratio images were overlaid over the 485/20 nm images of MDMs from the same time sequence, which had been false-colored white. MTrackJ plugin manually tracked the CD4^+^ T cells in each time sequence, which were measured with a circular point size of 20 pixels. Tracks of <5 min duration were immediately excluded. The level of T cell intracellular calcium (340:380 nm ratio) for every T cell in each time sequence was plotted over time, defining calcium signatures.

### Motility data analysis

MATLAB (The MathWorks Inc.) was used to manipulate.csv outputs from MTrackJ to generate the following thirteen measures of T cell behavior: mobile fraction (fraction of track where cell is moving >2 µm/min); track duration [s]; t_max 340:380_ [s] (time of max 340:380 ratio); % track length_max 340:380_ (percentage of track length at which t_max 340:380_ occurs); returns to baseline (R2B; number of returns to within 20% of the baseline, defined as the median value of ten tracks with lowest 340:380 range); min_340:380_ (minimum 340:380 value of the track); max_340:380_ (maximum 340:380 value of the track); median_340:380_ (median 340:380 value of the track); area under curve (AUC) _340:380_ (area between the 340:380 curve and the baseline/track duration); length_ratio_ (track length [µm] pre t_max 340:380_/track [µm] length post t_max 340:380_); speed_ratio_ (speed [µm/s] pre t_max 340:380_/speed [µm/s] post t_max 340:380_); meandering index (MI = Euclidean distance/track length)_ratio_ (MI pre t_max 340:380_/MI post t_max 340:380_); Euclidian distance_ratio_ (displacement [µm] from track origin to t_max 340:380_\displacement [µm] from t_max 340:380_ to track end). Tracks with fewer than three datapoints before or after t_max 340:380_ were excluded. For a comparison population of non-signalling cells, tracks from T cells with max. 340:380 ≤ 1 were selected according to the closest match of t_max 340:380_ to T cells with max. 340:380 ≥ 1.

### Correlative imaging of live and fixed MDM-CD4^+^ T cell conjugates

Live cell imaging of MDM-CD4^+^ T cell interactions was performed as described, but with CFSE-labelled MDMs seeded in a glass-bottomed 35 mm dish (MatTek corporation) and pulsed with 1 µg/ml sAgs. A mark was made on the base of the dish to facilitate orientation and location of a nearby region. Live cell imaging of the region took place for a total of 30 min, directly after which the sample was fixed in 3% PFA for 20 min, permeabilized and immunolabeled with anti-CD3 (OKT3) as described below. Guided by the mark, the original field of view was located on a confocal fluorescence microscope and z-stacks of the CD3 distribution of all remaining T cells in the region were acquired. The position of each T cell within the region was used to match the z-stack of a given T cell to the calcium signature it demonstrated prior to fixation.

### Classification of CD4^+^ T cell intracellular calcium levels

Population-level analysis: The maximum 340:380 nm ratio was used as a high-throughput, global readout of the extent of intracellular calcium signaling exhibited by each CD4^+^ T cell over the timeframe of the live-cell imaging assay. Where indicated, a max 340:380 nm threshold of 1 was utilized in order to exclude the large number of T cells that only underwent basal calcium signaling.

Single-cell analysis: The different calcium signatures were qualitatively classified in a blinded manner into four groups defined by *‘flat’*: no rise or sharp spikes above baseline calcium level; *‘transient’*: a rise in calcium followed by one or more returns to baseline over the course of the assay; *‘low’*: a rise in calcium that neither returned to baseline nor fulfilled the acceptance criteria of the *‘high’* group; *‘high’*: a calcium onset rising above a 340:380 ratio of 1.5 that is sustained above 1 for the duration of the experiment or, alternatively, a calcium onset exceeding a 340:380 ratio of 2.

### MDM-CD4^+^ T cell conjugate formation and immunolabeling for confocal fluorescence microscopy

2 × 10^5^ MDMs (day 8) were seeded onto 10 mm diameter glass coverslips and allowed to adhere for 24 h at 37 °C, 5% CO_2_. MDMs were pulsed, or not, with 1 µg/ml sAgs cocktail for 2 h, washed with medium, before autologous CD4^+^ T cells were added at a T cell: MDM ratio of 2:1 and interactions synchronized by centrifugation (100 × g for 1 min). MDMs and CD4^+^ T cells were co-incubated for 30 min, 37 °C, 5% CO2, then fixed with 3% PFA for 20 min at RT. Coverslips were washed and quenched with 50 mM NH_4_Cl in PBS for 20 min at RT. They were either left intact or permeabilized by including 0.05% Saponin in all subsequent labeling and wash solutions. For staining, Fc receptors were blocked with 30 µg/ml human IgG (hIgG) in PBS containing 1% FBS (PBS-FBS) for 30 min. Coverslips were incubated for 60 min at RT with the relevant primary antibodies or the corresponding isotype controls diluted at 5 µg/ml in PBS-FBS with 5 µg/ml hIgG. Samples were then washed and incubated for 60 min with 4 µg/ml GAM IgG1 AlexaFluor488 and IgG2a AlexaFluor647, or anti-sheep AlexaFluor488 secondary antibodies in PBS-FBS with 5 µg/ml hIgG, as indicated. Following further washes, samples were labeled with 1 µg/ml DAPI in PBS for 5 min before coverslips were mounted in mowiol. Individual confocal sections or z-stacks images were acquired to analyze the distribution of cell markers, as indicated. Controls showed no negligible binding of isotypes controls and no cross-reactivity. Experiments were performed with cells from N ≥ 3 donors.

### Analysis of marker accumulation to the MDM-CD4^+^ T cell interface

Bright-field images were used as a position guide in order to manually trace the plasma membrane of cells (traces of 10 pixels in width) over the following regions: (i) the MDM-CD4^+^ T cell interface; (ii) the CD4^+^ T cell surface not in contact with the MDM. The MFI of cell surface labelling within each region was measured. A ratio of surface marker accumulation at the interface was calculated by dividing the interface MFI by the MFI measured across the remaining T cell surface. For permeablized cells, 50 × 50 pixel square (3.37 × 3.37 µm) regions of interest (ROI) were drawn at four positions: the interface, opposite the interface (away), and either side of the T cell, to include intracellular staining in the quantification. The MFI within each ROI was expressed as a percentage of the sum MFI of all four ROIs. A measure of side-to-side and front-to-back polarity was determined and plotted together on x and y axes respectively, to graphically indicate the overall polarization of individual markers when interacting with a MDM.

### Statistical analyses

Data were analyzed with GraphPad PRISM v5.03 and normality determined using D’Agostino-Pearsons test before statistical comparison with the appropriate test, as indicated. *P*-values of <0.05 were considered significant: *P < 0.05, **P < 0 0.01, and ***P < 0.005. PCA and LDA were performed on scaled data from all samples, using MATLAB algorithms and R^48^, respectively. LDA was implemented in the package MASS^28^. Figures were produced using ggplot2^49^ and ggbiplot^50^. Individual scripts are provided as supplemental files.

### Data Availability

Raw datasets generated during and/or analyzed during the current study are available from the corresponding author on reasonable request.

## Electronic supplementary material

Movie 1

Movie 2

Movie 3

Movie 4

Movie 5

Supplemental file

Calcium measures dataset
