# Supplementary material for: A correlative and quantitative imaging approach enabling characterization of primary cell-cell communication: Case of human CD4+ T cell-macrophage immunological synapses
Source: Sci Rep. 2018 May 22;8:8003. doi: 10.1038/s41598-018-26172-3 (PMC5964238; doi:10.1038/s41598-018-26172-3)
Supplement: Supplementary file 6 — Supplemental file [file 41598_2018_26172_MOESM6_ESM.pdf]

A correlative and quantitative imaging approach enabling characterization of primary cell-cell communication: Case of human CD4⁺ T cell-macrophage immunological synapses.

Richard Kasprowicz, Emma Rand, Peter J O’Toole and Nathalie Signoret

Supplementary information

Supplemental Methods:

Epifluorescence for live calcium imaging:

Live imaging was performed on a Nikon Eclipse TE200 wide-field inverted fluorescence microscope (Nikon Instruments) equipped with a Plan Fluor 10X/0.3 NA objective, mercury lamp, an automated filter-wheel controller (OptiScan^{TM} II; Prior Scientific), a custom-built 37°C incubator box, and SimplePCI software (Hamamatsu Corporation). The focal plane was set to the position of the adherent MDMs and imaging was initiated immediately after fura-2-loaded $CD4^+$ T cells had been introduced into the imaging chamber at a rate of ~33 µl/s using a custom-designed syringe pump. The sample was excited via 340 nm, 380 nm and 485/20 nm filters at 10 s intervals for a total duration of 20 min. Images were captured via a FITC 510-560 band-pass emission filter using a Rolera-XR Fast 1294 camera (Q-Imaging).

Confocal microscopy:

We used a Zeiss LSM710 confocal fluorescence microscope with ZEN 2009 software and a Plan-Apochromat 63x/1.4 NA Oil objective. The X, Y pixel resolution of each defined region and the Z step size was optimized to achieve Nyquist sampling. Images of DAPI, AlexaFluor-488 and AlexaFluor-647 fluorophores were acquired sequentially using a diode laser (405nm) 30mW, Ar ion laser (488nm) 30mW and HeNe-laser (633nm) 5mW. Gain (detector sensitivity) was adjusted so as not to saturate pixels within the 8-bit dynamic range of the image whilst giving no detectable signal in isotype-matched controls. Spectral detector emission ranges were set at 410-585nm, 490-626nm, and 638-755nm for the aforementioned illumination sources, respectively.

Multivariate analysis:

Table of calcium and motility measures from all samples are available as supplemental dataset (**FullCalciumTable.csv** file). Details of scripts used for PCA and LDA in MATLAB and R, respectively are provided bellow:

PCA analysis on calcium & Motility measures in MATLAB:

```
%uses the 'fullSubtypes' file without the header as subtypes

%uses the 'fullCalciumTable' file without the header as calciumTable
%(header as calciumTableHeader)

%THEN RUN PCA (the data has already been z-scored)
%[coeff,score,latent,tsquared,explained,mu] = pca(calciumSignatures);

%these output variables relate to tables of the same name (e.g. score.csv)

%***************************************************************************
%scree plot
figure()
pareto(explained)
title('All Donors')
xlabel('Principal Component')
ylabel('Variance Explained (%)')
%***************************************************************************
%Visualize both the orthonormal principal component coefficients for each
%variable and the principal component scores for each observation in a single plot.

figure('Color','w');
set(0, 'defaultTextFontSize',11, 'defaultTextFontWeight', 'bold')
hbi = biplot(coeff(:,1:3), ...
   'Scores', score(:,1:3), ...
   'VarLabels', calciumTableHeader, ...
   'ObsLabels', num2str(subtypes), ...
   'markersize', 5, 'LineWidth', 4);
title('Bi-Plot: All Donors', 'FontSize', 24);
```

```
xlabel(['PC1-(' num2str(round(explained(1))) '%)'], 'FontSize', 18);
ylabel(['PC2-(' num2str(round(explained(2))) '%)'], 'FontSize', 18);
zlabel(['PC3-(' num2str(round(explained(3))) '%)'], 'FontSize', 18);
pbaspect([1 1 1]);
box on;
set(gca,'XLim',[-0.27 0.6],'YLim',[-0.27 0.6],'ZLim',[-0.45 0.65])


% Manipulate plot colors
for ii = 1:length(hbi)-length(subtypes)
    set(hbi(ii), 'Color', [0.2 0.2 0.2], 'Visible', 'on');
end
for ii = length(hbi)-length(subtypes):length(hbi)
    userdata = get(hbi(ii), 'UserData');
    if ~isempty(userdata)
        if subtypes(userdata) == 0
            set(hbi(ii), 'MarkerFaceColor', 'none', 'MarkerEdgeColor', [0 0.69 0.94], 'Marker', 'o', 'LineWidth', 0.001, 'Visible', 'on');
        elseif subtypes(userdata) == 1
            set(hbi(ii), 'MarkerFaceColor', 'none', 'MarkerEdgeColor', [1 0.31 0.31],'Marker', 'o', 'LineWidth', 0.001, 'Visible', 'on');
        elseif subtypes(userdata) == 2
            set(hbi(ii), 'MarkerFaceColor', 'none', 'MarkerEdgeColor', [0.57 0.82 0.31],'Marker', 'o', 'LineWidth', 0.001, 'Visible', 'on');
        elseif subtypes(userdata) == 3
            set(hbi(ii), 'MarkerFaceColor', 'none', 'MarkerEdgeColor', [0.44 0.19 0.63],'Marker', 'o', 'LineWidth', 0.001, 'Visible', 'on');
        elseif subtypes(userdata) == 4
            set(hbi(ii), 'MarkerFaceColor', [0 0.69 0.94], 'MarkerEdgeColor', [0 0.69 0.94], 'Marker', 'o', 'LineWidth', 0.001, 'Visible', 'on');
        elseif subtypes(userdata) == 5
            set(hbi(ii), 'MarkerFaceColor', [1 0.31 0.31], 'MarkerEdgeColor', [1 0.31 0.31],'Marker', 'o', 'LineWidth', 0.001, 'Visible', 'on');
        elseif subtypes(userdata) == 6
            set(hbi(ii), 'MarkerFaceColor', [0.57 0.82 0.31], 'MarkerEdgeColor', [0.57 0.82 0.31],'Marker', 'o', 'LineWidth', 0.001, 'Visible',
'on');
        elseif subtypes(userdata) == 7
            set(hbi(ii), 'MarkerFaceColor', [0.44 0.19 0.63], 'MarkerEdgeColor', [0.44 0.19 0.63],'Marker', 'o', 'LineWidth', 0.001, 'Visible',
'on');
        end
    else
        set(hbi(ii), 'Color', 'k', 'Visible', 'off');
    end
end

view([0 0 90]) % PC1 vs PC2
%view([90 0 0]) % PC2 vs PC3
%view([0 -90 0]) % PC1 vs PC3
```

Subtyping cells on calcium profile using R:

```
# The data in FullCalciumTable.csv are from an investigation of
# calcium profiles for cell subtypes

# The data are organised
# 'data.frame':     1391 obs. of  13 variables:
#   $ Track.duration              : int  770 870 880 850 790 780 660 510 590 360 ...
# $ Min.340.380                  : num  0.167 0.286 0.273 0.357 0.333 ...
# $ Max.340.380                  : num  0.438 0.6 0.455 0.5 0.467 ...
# $ t_.Max.340.380.              : int  1030 1150 610 970 1050 710 580 990 1120 1150 ...
# $ X..track.length_.Max.340.380.: num  79.2 95.4 34.1 74.1 82.3 ...
# $ Median.340.380               : num  0.368 0.429 0.391 0.417 0.389 ...
# $ Length_.ratio.               : num  4.72 19.5 1.05 1.95 4.29 ...
# $ Euclidean_.ratio.            : num  -8.89 -2.55 631.65 -1.42 13.03 ...
# $ MI_.ratio.                   : num  1.884 0.131 603.19 0.733 3.036 ...
# $ Speed_.ratio.                : num  0.786 0.855 1.03 0.466 0.619 ...
# $ AUC_.340.380.                : num  0.00574 0.05259 0.01649 0.02941 0.01292 ...
# $ Baseline.returns             : num  0 0.01609 0 0.00471 0.00127 ...
# $ Mobile.fraction              : num  0.61 0.494 0.455 0.447 0.608 ...

# the cell type labels are in FullSubtypes.csv
# celltype labels, numeric code 0,1,2,4,5,6,7 note no type 3


##########################################################################
#                        Set up                              #
##########################################################################

# working directory (in my case, yours will differ)
setwd("~/rhelp")

# packages
library(ggplot2)
# H. Wickham. ggplot2: Elegant Graphics for Data Analysis.
# Springer-Verlag New York, 2009.
```

```
library(ggbiplot)
# Vincent Q. Vu (2011). ggbiplot: A ggplot2 based biplot. R package
# version 0.55.
# http://github.com/vqv/ggbiplot

library(MASS)
# Venables, W. N. & Ripley, B. D. (2002) Modern Applied Statistics
# with S. Fourth Edition. Springer, New York. ISBN 0-387-95457-0

# palette
palette <- c("deepskyblue", "red", "chartreuse3", "darkorchid4")


#####################################################################
#                     Import and tidy data                          #
#####################################################################

# celltype labels, numeric code 0,1,2,4,5,6,7 note no type 3
fullsubtypes <- read.csv("FullSubtypes.csv")

# explore
str(fullsubtypes)
table(fullsubtypes$subtypes)

# make subtype a factor
fullsubtypes$fsubtypes <- factor(fullsubtypes$subtypes)

# add a factor for subtypes where 0 and 4 are flat,
# 1 and 5 are transient, 2 and 6 and low and 7 is high
fullsubtypes$fsubtypes2[fullsubtypes$fsubtypes == "0"] <- "flat"
fullsubtypes$fsubtypes2[fullsubtypes$fsubtypes == "4"] <- "flat"

fullsubtypes$fsubtypes2[fullsubtypes$fsubtypes == "1"] <- "transientient"
fullsubtypes$fsubtypes2[fullsubtypes$fsubtypes == "5"] <- "transientient"

fullsubtypes$fsubtypes2[fullsubtypes$fsubtypes == "2"] <- "low"
fullsubtypes$fsubtypes2[fullsubtypes$fsubtypes == "6"] <- "low"

fullsubtypes$fsubtypes2[fullsubtypes$fsubtypes == "7"] <- "high"

fullsubtypes$fsubtypes2 <- factor(fullsubtypes$fsubtypes2,
                                  levels = c("flat", "transientient", "low", "high"))
levels(fullsubtypes$fsubtypes2)

# import measured variables
fullcalciumtable <- read.csv("FullCalciumTable.csv")

# boxplot of measurements to assess whether scaling is needed
# and indicate potential outliers

boxplot(fullcalciumtable)
# scaling is required
# euclidean ration and mi ration look to have extreme outliers

#####################################################################
#       Explore scaled with PCA - outliers included                 #
#####################################################################


# PCA
pca <- prcomp(fullcalciumtable, scale. = TRUE)
summary(pca)
# do seem to need 9 components to capture 95% of the variation
# PC1 = 25.4%, PC2 = 17.2%, PC3 = 13.6%, cummulative in 3 is 56%

# dataframe of PCA scores with subtype labels (4 categories)
pca_labelled <- data.frame(pca$x, subtypes = fullsubtypes$fsubtypes2)
# PC1 and PC2
ggplot(data = pca_labelled, aes(x = PC1, y = PC2, color = subtypes)) +
  geom_point()
# PC1 and PC3
ggplot(data = pca_labelled, aes(x = PC1, y = PC3, color = subtypes)) +
  geom_point()
# PC2 and PC3
ggplot(data = pca_labelled, aes(x = PC2, y = PC3, color = subtypes)) +
  geom_point()

# A fancier biplot
# PC1 and PC2
pcabiplot <- ggbiplot(pca, choices = c(1, 2), groups = pca_labelled$subtypes,
        ellipse = TRUE,
```

```
        ellipse.prob = 0.95,
        varname.size = 4,
        varname.adjust = 2.5) +
  scale_colour_manual(values = palette, name = "Classification") +
  theme(panel.background = element_rect(fill = "white", color = "black"))+
  theme(axis.line.x = element_line(color = "black"),
       axis.line.y = element_line(color = "black")) +
  theme(legend.key=element_blank())

# send image to file
png(filename = "pcabiplot.png", width = 500, height = 500)
plot(pcabiplot)
dev.off()

######################################################################
#  Linear discriminant analysis (LDA) - outliers included            #
######################################################################

# perform LDA
ldaout <- lda(fullcalciumtable, fullsubtypes$fsubtypes2)

# To determine how well the subtypes are predicted
# Note: testing and training on 100% data

#
pldaout <- predict(object = ldaout,
                   newdata = fullcalciumtable)

# how many predicted classes are the same as the actual
# classes, over all
overallcorrect <- table(pldaout$class == fullsubtypes$fsubtypes2)
overallcorrect[2]/sum(overallcorrect)*100
# FALSE  TRUE
# 214  1177
# 84.6% are correctly predicted

# correct per class
perclasscorrect <- table(predicted = pldaout$class,
                         actual = fullsubtypes$fsubtypes2)
#          actual
# predicted flat transientient low high
# flat       924   132  15    0
# transientient        2   134  33    0
# low          1    24  78    1
# high         0     1   5   41

# % each class correctly predicted
flatcorrect <- round(perclasscorrect[1, 1] /
                       sum(perclasscorrect[,1]) * 100, 1)
transientcorrect <- round(perclasscorrect[2, 2] /
                      sum(perclasscorrect[,2]) * 100, 1)
lowcorrect <- round(perclasscorrect[3, 3] /
                       sum(perclasscorrect[,3]) * 100, 1)
highcorrect <- round(perclasscorrect[4, 4] /
                       sum(perclasscorrect[,4]) * 100, 1)
# flatcorrect 99.7
# transientientcorrect 46
# lowcorrect 59.5
# highcorrect 97.6


# we add the labels of the original and class prediction
ldaout_labelled <- data.frame(pldaout$x,
                              subtypes = fullsubtypes$fsubtypes2,
                              pldaout$class)

# add a variable which indicates if predictions were correct
indic <- pldaout$class == fullsubtypes$fsubtypes2
ldaout_labelled <- data.frame(ldaout_labelled, correct = indic)

# biplot with LD 1 and 2
ldabiplot <- ggbiplot(ldaout, choices = c(1, 2),
        groups = ldaout_labelled$subtypes,
        ellipse = TRUE,
        ellipse.prob = 0.95,
        varname.size = 4,
        varname.adjust = 2) +
  xlab("LD1") +
  ylab("LD2")+
  xlim(-5, 20) +
  scale_colour_manual(values = palette, name = "Classification") +
  theme(panel.background = element_rect(fill = "white", color = "black"))+
```

```
  theme(axis.line.x = element_line(color = "black"),
        axis.line.y = element_line(color = "black")) +
  theme(legend.key=element_blank())
# send image to file
png(filename = "ldabiplot.png", width = 500, height = 500)
plot(ldabiplot)
dev.off()

# figure with correctly (TRUE and incorrectly predicted classes)
pc <- c(flatcorrect, transientcorrect, lowcorrect, highcorrect)
pc <- paste0(pc ,"%")
dat_text <- data.frame(label = c(NA, NA, NA, NA,
                                 pc),
                       pldaout.class = levels(ldaout_labelled$subtypes),
                       correct = rep(c(FALSE, TRUE), each = 4))
classplot <- ggplot() +
geom_point(data = ldaout_labelled,aes(x = LD1, y= LD2, color = subtypes), size = 2) +
  facet_grid(correct ~ pldaout.class) +
  scale_colour_manual(values = palette, name = "Classification") +
  theme(panel.background = element_rect(fill = "white", color = "black"),
        strip.background =element_rect(fill = "white", color = "black"))+
  theme(axis.line.x = element_line(color = "black"),
        axis.line.y = element_line(color = "black")) +
  geom_text(data = dat_text, aes(x = 2, y = -6, label = label)) +
  theme(legend.key=element_blank())

# send image to file
png(filename = "classplot.png", width = 800, height = 400)
plot(classplot)
dev.off()

#########################################################################
#  Linear discriminant analysis (LDA) - outliers excluded            #
#########################################################################

# exclude outliers
boxplot(fullcalciumtable2)
# decide to exclude those above 2000
fullcalciumtable2 <- fullcalciumtable[fullcalciumtable$Euclidean_.ratio. < 2000,]
fullcalciumtable2 <- fullcalciumtable2[fullcalciumtable2$MI_.ratio. < 2000,]
fullcalciumtable2 <- fullcalciumtable2[complete.cases(fullcalciumtable2),]

# exclude the rows with those labels from the subtypes column too
fullsubtypes2 <- fullsubtypes[fullcalciumtable$Euclidean_.ratio. < 2000,]
fullsubtypes2 <- fullsubtypes2[fullcalciumtable2$MI_.ratio. < 2000,]
fullsubtypes2 <- fullsubtypes2[complete.cases(fullsubtypes2),]

# perform LDA
ldaout2 <- lda(fullcalciumtable2, fullsubtypes2$fsubtypes2)

# To determine how well the subtypes are predicted
# Note: testing and training on 100% data

#
pldaout2 <- predict(object = ldaout2,
                   newdata = fullcalciumtable2)
levels(pldaout2$class)
# how many predicted classes are the same as the actual
# classes, over all
overallcorrect2 <- table(pldaout2$class == fullsubtypes2$fsubtypes2)
overallcorrect2[2]/sum(overallcorrect2)*100
# FALSE  TRUE
# 208  1180
# 85% are correctly predicted, very slightly better

# correct per class
perclasscorrect2 <- table(predicted = pldaout2$class,
                        actual = fullsubtypes2$fsubtypes2)
#           actual
# predicted flat transient low high
# flat       924   129  15    0
# transient        2   137  32    0
# low          0    24  79    1
# high         0     0   5   40

# % each class correctly predicted
flatcorrect2 <- round(perclasscorrect2[1, 1] /
                     sum(perclasscorrect2[,1]) * 100, 1)
transientcorrect2 <- round(perclasscorrect2[2, 2] /
                    sum(perclasscorrect2[,2]) * 100, 1)
lowcorrect2 <- round(perclasscorrect2[3, 3] /
                     sum(perclasscorrect2[,3]) * 100, 1)
```

```
highcorrect2 <- round(perclasscorrect2[4, 4] /
                    sum(perclasscorrect2[,4]) * 100, 1)

# we add the labels of the original and class prediction
ldaout2_labelled <- data.frame(pldaout2$x,
                           subtypes = fullsubtypes2$fsubtypes2,
                           pldaout2$class)

# add a variable which indicates if predictions were correct
indic2 <- pldaout2$class == fullsubtypes2$fsubtypes2
ldaout2_labelled <- data.frame(ldaout2_labelled, correct = indic2)

# biplot with LD 1 and 2
ldabiplot2 <- ggbiplot(ldaout2, choices = c(1, 2),
                    groups = ldaout2_labelled$subtypes,
                    ellipse = TRUE,
                    ellipse.prob = 0.95) +
  xlab("LD1") +
  ylab("LD2")+
  scale_colour_manual(values = palette, name = "Classification") +
  theme(panel.background = element_rect(fill = "white"))+
  theme(axis.line.x = element_line(color = "black"),
        axis.line.y = element_line(color = "black")) +
  theme(legend.key=element_blank())
# send image to file
png(filename = "ldabiplot2.png", width = 500, height = 500)
plot(ldabiplot2)
dev.off()

# figure with correctly (TRUE and incorrectly predicted classes)
pc2 <- c(flatcorrect2, transientcorrect2, lowcorrect2, highcorrect2)
pc2 <- paste0(pc2 ,"%")
dat_text2 <- data.frame(label = c(NA, NA, NA, NA,
                              pc),
                    pldaout2.class = levels(ldaout2_labelled$subtypes),
                    correct = rep(c(FALSE, TRUE), each = 4))
classplot2 <- ggplot() +
  geom_point(data = ldaout2_labelled,aes(x = LD1, y= LD2, color = subtypes), size = 2) +
  facet_grid(correct~pldaout2.class) +
  scale_colour_manual(values = palette, name = "Classification") +
  theme(panel.background = element_rect(fill = "white", color = "black"),
        strip.background =element_rect(fill = "white", color = "black"))+
  theme(axis.line.x = element_line(color = "black"),
        axis.line.y = element_line(color = "black")) +
  geom_text(data = dat_text2, aes(x = 1, y = -6, label = label)) +
  theme(legend.key=element_blank())

# send image to file
png(filename = "classplot2.png", width = 800, height = 400)
plot(classplot2)
dev.off()

# conclusion: removing outliers doesn't really have an effect
```

Supplemental figures and legends:

Fig. S1: Comparing calcium signals of T cells perfused on MDMs ± SEE. Disparate responses of cells isolated from two different donors were found during statistical comparison of the distributions of max 340:380 ratio values between T cells with unpulsed or SEE-pulsed autologous MDMs. A donor variability related to the detection of all signaling T cells in mixed cells populations for a low frequency of conjugates formed (8%), as indicated by the flow cytometry experiment.

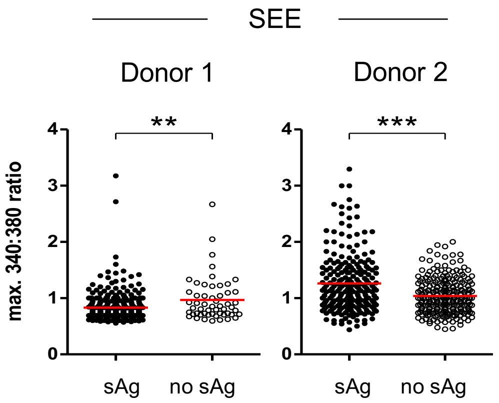

Fig. S2: Motility parameters in absence of sAgs: Each point represents an individual cell measured from donor (n=3) samples in absence of sAgs [Mean, x; Bars: median ± interquartile range. Statistical analysis: Mann Whitney U test].

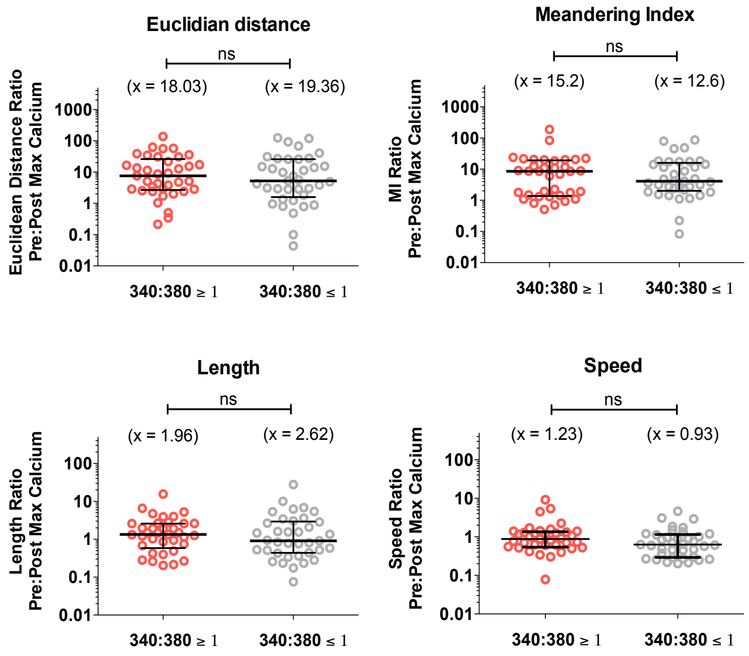

Fig. S3: Correlative imaging analysis of signal productive MDM-CD4⁺ T cell synapses. A) Following calcium live imaging, fixation and immunolabeling of samples removed T cells that are not strongly adhered to MDMs, increasing the incidence of MDM-CD4⁺ T cell conjugates with high and low calcium profiles analyzed. **B)** Planar view of IS detected by confocal microscopy and showing the characteristic concentric and segregated organization of ICAM-1 and CD3 on opposite sides of the MDM-CD4⁺ T cell interface, respectively; scale bar = 2 μm.

A

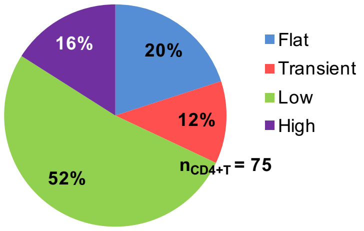

B

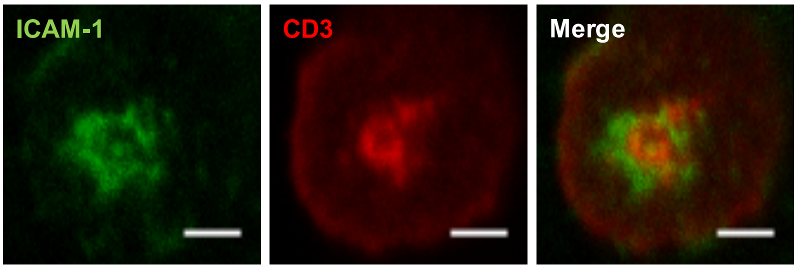

Fig. S4: CCR5 expression on blood-isolated and PHA/IL-2 activated CD4$^+$ T cells. Only a proportion of isolated CD4$^+$ T cells express CCR5 (Day 1), and CCR5 expression did not change with PHA/IL-2 treatment (Day 9), unlike what we previously described for total blood T cells (Fox et al. 2015, J. Leuk. Biol. 98: 59-71). Representative histograms for two specific anti-CCR5 monoclonal antibodies are shown overlaid on the relevant isotype control (shaded grey). The box plots show the percentage of CCR5 positive cells, statistical comparison was performed using a paired Student's t-test (ns= non-significant).

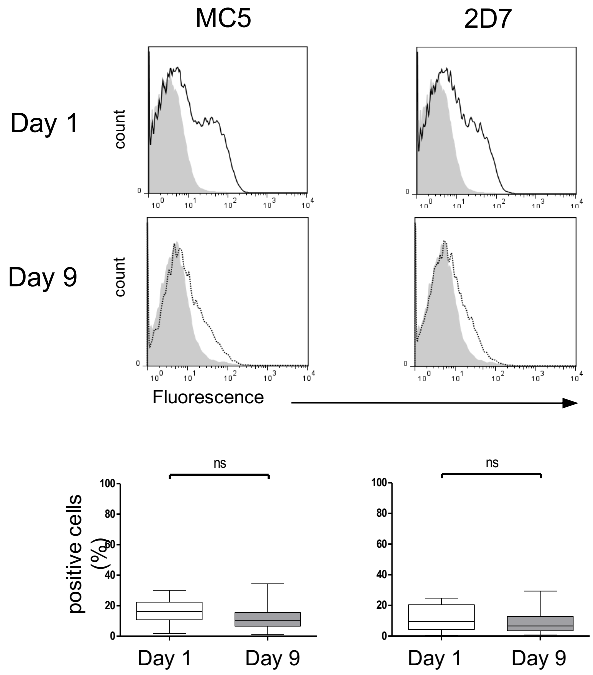

Fig. S5: Colocalization of TGN or TfR with CCR5. MDM-CD4+ T cell conjugates were formed, fixed and immunolabeled for (**a**) TGN and CCR5 followed by anti-sheep IgGs-488 and GAM IgG2a-647 or (**b)** TfR and CCR5 followed by GAM-IgG1-488 and GAM-IgG2a-647 secondary antibodies according to the method detailed in main manuscript. Three examples of CCR5$^+$ conjugates formed ± sAgs are shown, which are representative of z-stacks acquired from two or three donors. For each example, individual slices of the z-stack are shown over the region of CCR5 intracellular accumulation at the MDM-CD4+ T cell interface. Within these slices the dotted box indicates the region where TGN or TfR and CCR5 are shown as separate greyscale images. Scale bar, 5 µm.

a

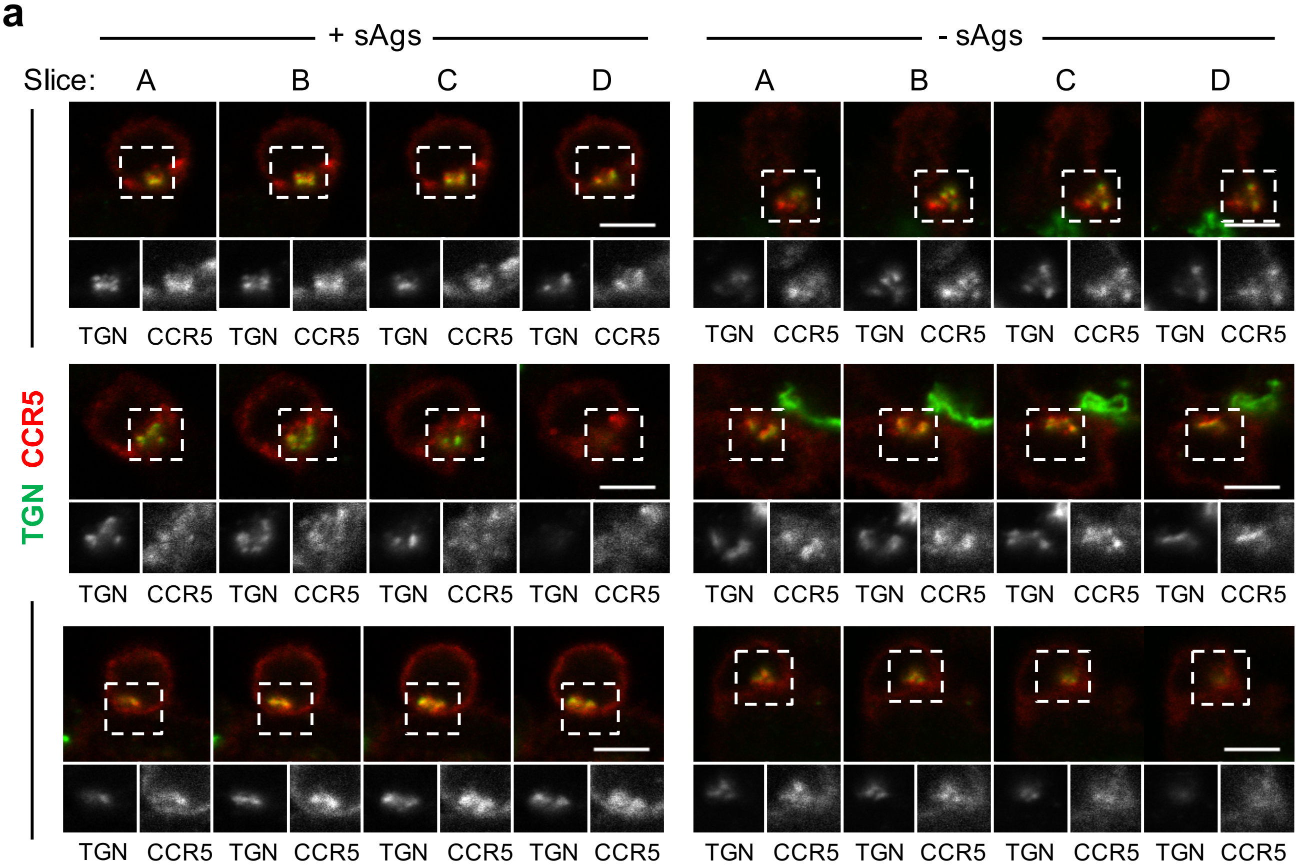
+ sAgs
- sAgs
Slice: A B C D
A B C D
TGN CCR5
TGN CCR5 TGN CCR5 TGN CCR5 TGN CCR5
TGN CCR5 TGN CCR5 TGN CCR5 TGN CCR5

b

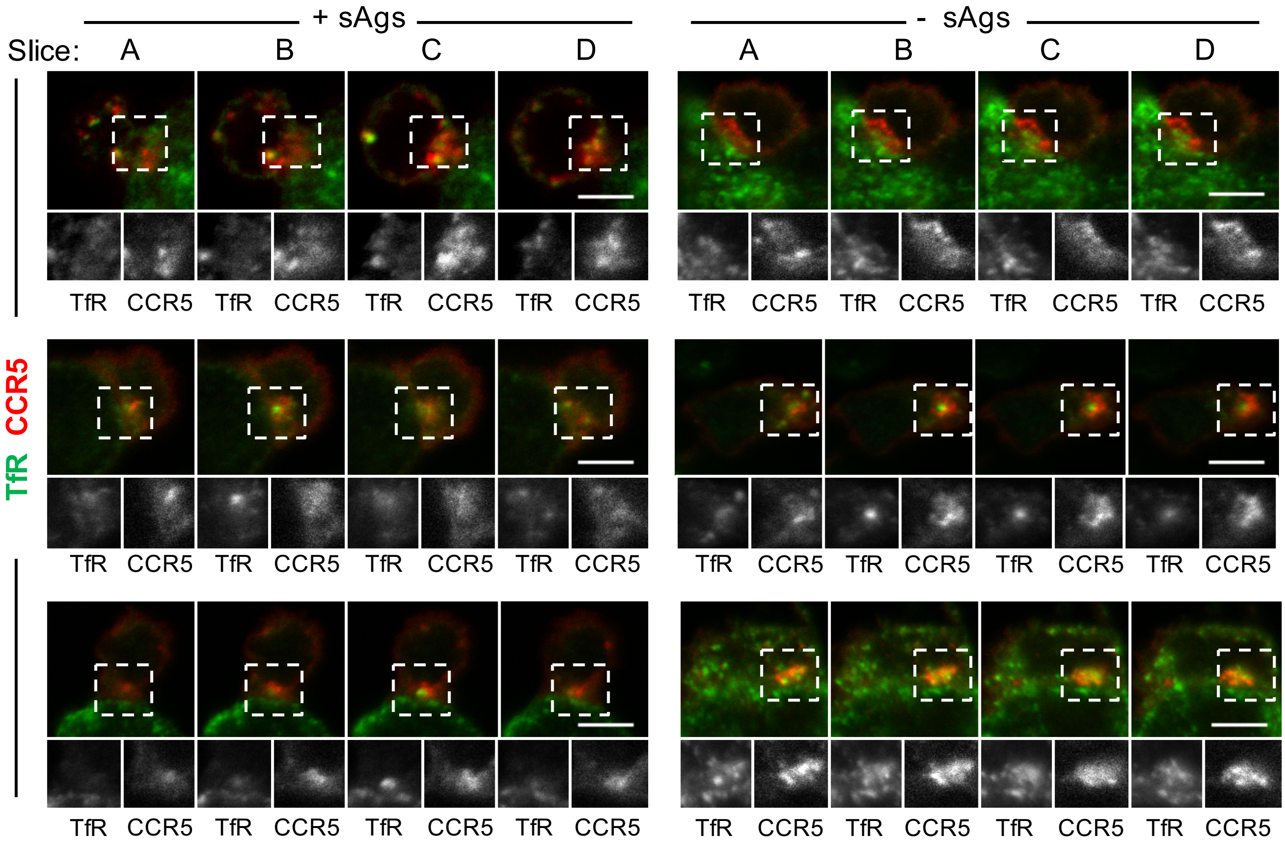
+ sAgs
- sAgs
Slice: A B C D
A B C D
TfR CCR5
TfR CCR5 TfR CCR5 TfR CCR5 TfR CCR5
TfR CCR5 TfR CCR5 TfR CCR5 TfR CCR5

Movies legends:

Mov. 1: Calcium response representative movies from Donor C

Mov. 2: Calcium response representative movies from Donor B

Mov. 3: Calcium response representative movies from Donor A

Mov. 4: Interplay between calcium flux and motility in individual $CD4^+$ cell – MDM interactions

Mov. 5: 3D bi-plot of first three principal components
